# From buds to shoots: insights into grapevine development from the Witch’s Broom bud sport

**DOI:** 10.1186/s12870-024-04992-y

**Published:** 2024-04-16

**Authors:** Eleanore J. Ritter, Peter Cousins, Michelle Quigley, Aidan Kile, Sunil K. Kenchanmane Raju, Daniel H. Chitwood, Chad Niederhuth

**Affiliations:** 1 Department of Plant Biology, Michigan State University, East Lansing, MI USA; 2 E. & J. Gallo Winery, Modesto, CA USA; 3 Department of Horticulture, Michigan State University, East Lansing, MI USA; 4 Center for Quantitative Imaging, Institute of Energy and the Environment, Penn State University, State College, PA USA; 5 Center for Genomics and Systems Biology, New York University, Manhattan, NY USA; 6 Department of Computational Mathematics, Science & Engineering, Michigan State University, East Lansing, MI USA; 7 Present Address: Corteva, Inc. Indianapolis, IN, USA

**Keywords:** Development, Grapevine, *Vitis vinifera*, Bud sport, Somatic mutations, Clonal propagation

## Abstract

**Background:**

Bud sports occur spontaneously in plants when new growth exhibits a distinct phenotype from the rest of the parent plant. The Witch’s Broom bud sport occurs occasionally in various grapevine (*Vitis vinifera*) varieties and displays a suite of developmental defects, including dwarf features and reduced fertility. While it is highly detrimental for grapevine growers, it also serves as a useful tool for studying grapevine development. We used the Witch’s Broom bud sport in grapevine to understand the developmental trajectories of the bud sports, as well as the potential genetic basis. We analyzed the phenotypes of two independent cases of the Witch’s Broom bud sport, in the Dakapo and Merlot varieties of grapevine, alongside wild type counterparts. To do so, we quantified various shoot traits, performed 3D X-ray Computed Tomography on dormant buds, and landmarked leaves from the samples. We also performed Illumina and Oxford Nanopore sequencing on the samples and called genetic variants using these sequencing datasets.

**Results:**

The Dakapo and Merlot cases of Witch’s Broom displayed severe developmental defects, with no fruit/clusters formed and dwarf vegetative features. However, the Dakapo and Merlot cases of Witch’s Broom studied were also phenotypically different from one another, with distinct differences in bud and leaf development. We identified 968–974 unique genetic mutations in our two Witch’s Broom cases that are potential causal variants of the bud sports. Examining gene function and validating these genetic candidates through PCR and Sanger-sequencing revealed one strong candidate mutation in Merlot Witch’s Broom impacting the gene GSVIVG01008260001.

**Conclusions:**

The Witch’s Broom bud sports in both varieties studied had dwarf phenotypes, but the two instances studied were also vastly different from one another and likely have distinct genetic bases. Future work on Witch’s Broom bud sports in grapevine could provide more insight into development and the genetic pathways involved in grapevine.

**Supplementary Information:**

The online version contains supplementary material available at 10.1186/s12870-024-04992-y.

## Background

Bud sports arise when a part of a plant, such as a lateral shoot, develops phenotypic differences from the rest of the parental plant. They typically arise when a somatic mutation occurs within a developing meristem and then spreads throughout the meristem and developing tissue [1]. Bud sports are known to arise sporadically in many perennial crops and can be an important source of novel phenotypes, having given rise to many plant cultivars widely grown today. They can be an especially important source of variation in difficult to breed perennial crops, such as grapevine (*Vitis vinifera*), which is challenging to breed due to high genetic heterozygosity and long regeneration times. As a result, beneficial bud sports in grapevines have often propagated to be grown as new varieties. For example, the variety Tempranillo Blanco first arose as a bud sport of Tempranillo Tinto and was clonally propagated to maintain its novel phenotype [2]. Bud sports are not always beneficial and sometimes detrimental to agricultural production, however, such bud sports provide natural mutants that can still be leveraged to study developmental traits that might otherwise not be possible [1].

Grapevines have unique development and physiology compared to many other crops and model systems. They are perennial plants that grow as lianas (also known as woody vines). This growth habit is enabled by tendrils, which are uncommon structures that allow them to climb as they grow. In addition, unlike many plants, their shoot tip does not terminate in an inflorescence, but instead contains an uncommitted primordium that allows the plants to continue growing from the tip [3]. Development within the buds of grapevines themselves is uniquely organized to ensure the successful production of leaves, tendrils, and inflorescences from the primordia. While tendril origin differs on a species basis, grapevine tendrils are modified inflorescences [3]. The switch from inflorescence development to tendril development occurs within the developing buds and is tightly regulated by a mixture of environmental conditions and hormones. Cytokinin signaling, high light, and high temperature promote inflorescence development while gibberellic acid (GA) signaling, low light, and low temperature promote tendril development [4]. Changes in hormones regulating these structures can have significant impacts on the ability of *V. vinifera* to sexually reproduce, even causing seed abortion [5]. However, understanding the regulatory and genetic components involved in grapevine development has proved challenging due to the difficulty of conducting genetic and molecular studies in grapevine.

Witch’s Broom (WB) is a bud sport that occurs spontaneously in multiple grapevine varieties. The WB phenotype involves prolific vegetative growth and limited to no production of flowers [6]. In contrast to wild type (WT), the WB bud sport does not easily root from cuttings, although the WB sport may be propagated by grafting. Similar WB bud sport phenotypes in other plant species are usually the result of pathogen infection, typically by phytoplasma [7–9]. However, genetic mutations have been shown to cause WB, as with the WB shoots in *Pinus sibirica* [10]. Cases of WB in grapevine are thought to have arisen through genetic causes and not pathogen infection. Instances of WB in grapevine do not spread within or between plants and have also occurred in plants that tested negative for pathogens. Therefore, WB bud sports in grapevines are thought to have arisen from genetic causes. As a result, the WB bud sport could be valuable for research, providing insight into an aspect of grapevine development and the genetic factors behind it, that would otherwise be near impossible to study. Here, we investigate both the phenotypic effects and the potential genetic underpinnings of two independent cases of the grapevine WB bud sport. Our results demonstrate that the WB bud sport impacts grapevine development from buds to shoots, but in distinct ways in the two cases we studied. Our work also suggests that the basis for the WB bud sports may result from mutations in different genes.

## Methods

### Plant material

Two independent cases of WB from two grapevine varieties, Merlot and Dakapo (*Vitis vinifera* L.), were sequenced and phenotyped alongside tissue from WT branches. The Merlot WT and WB samples were derived from the same plant, while the Dakapo WT and WB were derived from two separate plants.

The Merlot WB was identified as a bud sport on a vine of a Merlot plant in a commercial vineyard in Madera, California, USA that was planted in 1994 after being grafted to Harmony rootstock. The vineyard is trained to bilateral cordons, spur pruned, and planted with rows on an east/west orientation. The proband vine was observed in 2013 to have one arm with wild type shoots (the western arm) and one arm with WB shoots (the eastern arm). The plant material both collected and studied come from a mixture of the original proband Merlot vine and cuttings derived from it. The tissue samples used for short read sequencing were collected from the contrasting arms of the original proband Merlot vine for both Merlot WT and Merlot WB. Observations and tissue samples used for long read sequencing of the Merlot WB were from the WB arm of the original proband vine as well. In 2020, budwood was collected from the WB arm of the proband vine and bench grafted to Rupestris St. George rootstock by the commercial nursery Wonderful Nurseries in Wasco, California, USA. The Merlot WB cuttings used for imaging buds were collected (February 2021) from those grafted Merlot WB vines planted in Madera, California, USA in 2020. Cuttings from shoots on the Merlot WT arm of the proband vine were made in 2018 and rooted by the commercial nursery Greenheart Farms in Arroyo Grande, California, USA in individual pots. The vines resulting from those cuttings were planted in Madera, California, USA in 2018 and trained to bilateral cordons and spur pruned. Observations, tissue samples for long read sequencing, and cuttings of Merlot WT were collected from these planted cuttings from the proband vine.

The Dakapo WB was identified as a whole vine sport on a vine in a budwood increase block in Madera, California, USA that was planted in 2011. A budwood increase block is cultivated to provide propagation wood for grafting or cuttings rather than fruit for commercial production. The proband vine was observed in 2013 to demonstrate the WB phenotype, in contrast to nearby Dakapo WT vines of the same age in the same block. Budwood was collected from the proband vine and bench grafted in 2015 onto 140 Ruggeri rootstock by the commercial nursery Duarte Nursery in Hughson, California, USA. The grafted vines were planted in 2015. Observations and all samples of the Dakapo WB come from a single grafted vine. Observations and all samples of Dakapo WT are from the original Dakapo vines planted in the budwood increase block in 2011.

### Phenotyping of the WB bud sport

Shoot and leaf phenotyping was conducted on samples from field grown vines in Madera, California, USA in September 2021. Ten shoots were examined per accession (Merlot WB, Merlot WT, Dakapo WB, Dakapo WT). For WT vines, fertile (with fruit clusters) shoots from retained nodes were observed. For WB vines, shoots from retained nodes were observed. Retained nodes are nodes with dormant buds chosen by professional pruners during dormant pruning as the most likely to produce healthy shoots in an appropriate position during the subsequent growing season and ordinarily the shoots from retained are the most fruitful shoots on a grapevine. Lateral meristem presence and type was recorded for 16 nodes beginning at the basal end of the shoot. The lateral meristem choices were tendril, cluster, and shoot. If a scar was present indicating the loss of the lateral meristem, this was recorded as “scar” since the type of lateral meristem could not be determined by observation. Skipped nodes where no lateral meristem was present were recorded as a “skip”. The length of 16 internodes basal to those nodes was recorded. The maximum blade length, maximum blade width and the petiole length of five fully expanded undamaged leaves at or distal to the cluster zone were recorded from each of the ten shoots per accession.

### Leaf landmarking and analysis

Between 12 and 14 leaves were collected from six shoots per sample from plants in Madera, California, USA in June 2022. The sampled shoots grew from retained nodes. Leaves were pressed in an herbarium press at Madera, California, USA and shipped in the press to East Lansing, Michigan, USA for scanning and analysis. The leaves were scanned using a CanoScan 9000 F Mark II (Canon U.S.A., Inc) at 600 DPI. The leaves were landmarked manually by placing 21 landmarks from Bryson et al. [11] on leaf scans using ImageJ v1.53k [12]. Scans were saved as x- and y-coordinates in centimeters. The shoelace algorithm, originally described by Meister [13], was used to calculate leaf, vein, and blade areas using the landmarks. The landmarks were used as the vertices of polygons and the following formula, as described in Chitwood et al. [14], was used to then calculate the areas (where *n* represents the number of polygon vertices defined by the landmarked *x* and *y* coordinates):

$$\matrix{{{1 \over 2}\,\,\left| {{x_1}\,{y_2}\, + \,\,{x_2}\,{y_3}\, + \, \ldots \, + \,{x_{n - 1}}\,{y_n} + {x_n}\,{y_1}} \right.} \cr {\left. { - \,{x_2}\,{y_1}\, - \,{x_3}\,{y_2} - \, \ldots \, - \,{x_n}\,{y_{n - 1}}\, - \,{x_1}\,{y_n}} \right|} \cr }$$

To investigate changes in leaf shape between WT and WB leaves, a generalized Procrustes analysis and a principal components analysis (PCA) was performed using the shapes package v1.2.7 [15] in R v4.2.2 [16] and RStudio v2022.12.0.353 [17], with scaling and rotation. The shapes package v1.2.7 [15] in R and RStudio was also used to test for mean shape differences using a Hotelling’s *T*^*2*^ test.

### Data visualization

All plots were made in R using ggplot2 v3.4.2 [18] and arranged using cowplot v1.1.1 [19]. The R package ggsignif v0.6.4 was used to add significance bars to violin plots [20]. The R package ggnewscale v0.4.8 was used to plot distinct scales for WT and WB data when needed [21].

### Bud collection, dissecting, and imaging

Dormant grapevine cuttings were collected in Madera, California, USA in February 2021 and shipped overnight to East Lansing, Michigan, USA. The Dakapo WT and Merlot WT cuttings were between 6 and 7 mm in diameter, while the Dakapo WB and Merlot WB cuttings were between 4 and 5 mm in diameter. Cuttings were left at room temperature for 24–72 h before dissecting. Only live cuttings were used for bud dissection. The buds were dissected using a razor, slicing the buds vertically (parallel to the stem) until the primary, secondary, and tertiary buds could all be seen, but tendril primordia were still distinguishable. Buds were then imaged with a dissecting microscope.

Buds were also scanned to create 3D X-ray Computed Tomography (CT) reconstructions of internal anatomy. Three individual buds were scanned from Merlot WT cuttings, and four individual buds were scanned from cuttings for the other three samples. The scans were produced using the X3000 system (North Star Imaging) and the included efX software (North Star Imaging). The scans were taken at 75 kV and 100 µamps with a frame rate of 12.5 frames per second in continuous mode. 2880 projections and 2 frame averages were used. To obtain the maximum voxel size (4.5 μm), a subpix scan, which takes 4 scans at half a pixel distance and combines them to get approximately half the voxel size, was used (see scale, Fig. 5). The 3D reconstruction of the buds was computed with the efX-CT software. efX-View software was used to visualize 2D slices through the 3D reconstructions of the buds.

### Whole genome sequencing and alignment

Leaf tissue samples for sequencing were collected from all four accessions (Merlot WB, Merlot WT, Dakapo WB, Dakapo WT) in August 2018. DNA isolation was performed using the CTAB method as described in [22]. Library preparation for paired-end (PE) sequencing was performed as in [23] with slight modification and sequenced on a HiSeq 2500 (Illumina, Inc.) with 150 bp (bp) PE reads sequenced to 50-58X coverage. The reads were then prepared for downstream analysis, first using cutadapt v3.7 [24] to trim adapters and low-quality bases from the beginning and ends of reads with the following parameters: *q 20,20, --trim-n, -m 30*, and *-n 3*. The quality of the reads, both before and after trimming, were checked using FastQC v0.11.9 [25]. The trimmed reads were then mapped to the 12X.v2 grapevine reference genome assembly [26] using BWA-MEM v0.7.17 and the *-M* parameter [27]. Mapped reads were then prepared for variant calling by sorting them with Samtools v1.9 [28] and marking duplicate reads using Picard MarkDuplicates v2.15.0 [29]. The reads were then indexed using Samtools v1.9 [28], to enable use with downstream variant callers.

### Small variant calling and annotation

The GATK v4.0.12.0 [30] pipeline for short variant discovery was used to call small insertions and deletions (INDELs) and single nucleotide variants (SNVs) in the samples using the BAM files with marked duplicates [31]. GATK HaplotypeCaller was used to call SNVs and INDELs in the individual samples. The SNVs and INDELs were combined into one file and genotyped using GATK CombineGVCFs and GenotypeGVCFs, respectively. They were filtered with GATK VariantFiltration [31, 32], using the following filters: *MQ < 40.00, FS > 60.0, QD < 2.0, MQRankSum<-12.5*, and *ReadPosRankSum<-8.0*. These filters were chosen based on GATK’s recommendations for hard filtering germline short variants [33]. No additional filtering was done in order to avoid over-filtering and introducing false negatives that would reduce our likelihood of identifying casual variants. ANNOVAR was used to annotate the SNVs and INDELs [34] with the Genoscope 12X grapevine genome annotation [35] lifted to the 12X.v2 grapevine genome assembly [26] using liftoff [36] with the *-copies* parameter to minimize compatibility issues the newest grapevine genome annotation [26] had with downstream analyses.

### Long read sequencing

New tissue was collected for Oxford Nanopore Technologies (ONT) sequencing in July 2021. The tissue samples used were young leaves collected from actively growing shoot tips. The samples were frozen and shipped on dry ice overnight. The MSU Genomics Core extracted DNA from the samples and prepared the sequencing libraries. DNA was isolated from samples using a modified Qiagen Genomic-tip protocol (Qiagen) [37] with 5 mg lysing enzyme (0.5 mg/ml; L1412-5G; Sigma-Aldrich, Inc.), 5 mg Pectinase (0.5 mg/ml; P2401; Sigma-Aldrich, Inc.), and 500 µl Viscozyme L (5%; V2010-50; MilliporeSigma) added to the lysis buffer. Short read elimination was performed using the Circulomics Short Read Eliminator kit (formerly SS-100-101-01, now SKU 102-208-300; Pacific Biosciences). The size selected DNA was quantified using a Qubit 1.0 Fluorometer (Thermo Fisher Scientific) and the Qubit dsDNA BR (Broad Range) Assay (Q32853; Thermo Fisher Scientific). Barcoded sequencing libraries were then prepared using the Ligation Sequencing Kit 1D (SQK-LSK109; Oxford Nanopore Technologies) and Native Barcoding Expansion Kit (EXP-NBD104; Oxford Nanopore Technologies). The pooled libraries were then loaded on a PromethION FLO-PRO002 (R9.4.1; Oxford Nanopore Technologies) flow cell and sequenced on a PromethION24 (Oxford Nanopore Technologies), running MinKNOW Release 21.11.7 (Oxford Nanopore Technologies), to 19-31X coverage. Base calling, demultiplexing, and filtering were done using Guppy v5.1.13 (Oxford Nanopore Technologies) with the High Accuracy base calling model. Only reads with a mean Q-score ≥ 9 were kept.

### Long read alignment and structural variant calling

Adapters were trimmed from the ONT reads using Porechop v0.2.4 [38] with the following parameters: *--min_trim_size 5, --extra_end_trim 2, --end_threshold 80, --middle_threshold 90, --extra_middle_trim_good_side 2, --extra_middle_trim_bad_side 50*, and *--min_split_read_size 300*. NanoLyse v1.2.0 was used to remove ONT reads mapping to the lambda phage genome [39]. Low-quality reads and reads shorter than 300 base pairs (bp) were removed using NanoFilt v2.8.0 [39] with the following parameters: *-q 0* and *-l 300*. The quality of the trimmed and filtered reads was analyzed using FastQC v0.11.9 [25].

The ONT reads were mapped to the 12X.v2 grapevine reference genome assembly [26] using minimap2 v2.23-r1111 [40] two separate times with different parameters based on the needs of downstream programs. For use with sniffles v2.0.6 [41] to call structural variants (SVs), ONT reads were mapped with minimap2 v2.23-r1111 [40] and the following parameters: *-ax map-ont --MD*. The mapped reads were sorted with Samtools v1.9 [28]. Sniffles v2.0.6 [41] was first run on sorted mapped read files for all samples separately using the *--snf* parameter to generate .snf files for all samples. Sniffles v2.0.6 [41] was then run on the .snf files previously generated for WT and WB samples from the same variety, running Dakapo and Merlot separately, to create a VCF file with SVs.

The second version of ONT read mapping used minimap2 v2.23-r1111 [40] with parameters optimized for use with pbsv v2.8.0 (Pacific Biosciences) [42], an additional SV caller: *-a --MD --eqx -L -O 5,56 -E 4,1 -B 5 --secondary = no -z 400,50 -r 2k -Y*. Samtools v1.9 [28] was used to sort the mapped reads and add read groups. The sorted mapped read files were then used with pbsv v2.8.0 “discover”, running all samples separately to first discover signatures of structural variation and produce a .svsig file. A VCF file with SVs was then generated by running pbsv v2.8.0 “call” [42] with .svsig files for WT and WB samples from the same variety (with Dakapo and Merlot samples run separately) and the 12X.v2 grapevine reference genome assembly [26].

The SVs generated by sniffles and pbsv were first filtered to remove variants that did not pass the filters applied by the two variant callers. Sniffles performs filtering intrinsically by only keeping SVs 35 bp or longer in length, with a minimum number of supporting reads equal to or above 10% of the sequencing depth (2–3 reads for our samples). Sniffles also applies a “GT” tag for variants where the quality of the genotype is low, and SVs with this tag were filtered out. Pbsv performs filtering intrinsically by only keeping SVs 20 bp minimum in length, with at least 3 supporting reads across all samples and within samples, 1 supporting read per strand total across samples, and supporting reads above 20% of reads mapping to that site per sample. Pbsv also applies filters for variants near gaps in the reference genome or contig ends and for duplication variants with reads that do not fully span the region, which were all filtered out. For total structural variant counts by sample, the filtered VCF files from sniffles and pbsv were then merged using SURVIVOR v1.0.7 “merge” [43] to merge SVs identified by both programs that were greater than 30 bp long and within 300 bp of one another. To identify variants with genotypes specific to the WB samples and not present in WT, SnpSift v2017-11-24 [44] was used with the filtered VCF files to extract out variants either (a) only found in the WB sample (homozygous or heterozygous) or (b) homozygous in the WB sample and heterozygous in the WT sample. The VCF files filtered both by quality and SnpSift from sniffles and pbsv were then merged using SURVIVOR v1.0.7 “merge” [43] as described previously. Only SVs that met those two criteria for merging were used for downstream analysis. The genes overlapping with the merged SVs were identified using bedtools v2.30.0 “intersect” [45] and the Genoscope 12X grapevine genome annotation [35] lifted to the 12X.v2 grapevine genome assembly [26] using liftoff v1.6.2 [36] with the *-copies* parameter.

### Candidate gene analysis

To investigate a potential causal gene(s)/variant(s) for the WB budsport in grapevine, all genes with high impact SNVs/INDELs or SVs present in the WB samples and either (a) absent in WT (described as “novel” from hereinafter) or (b) heterozygous in WT but homozygous in WB, were investigated for gene function by looking into the functions of their closest Arabidopsis (*Arabidopsis thaliana*) ortholog. Variants matching either genotype criteria are described as “genotypically distinct” from hereinafter. In order to understand the putative functions of the genes with SNVs, INDELs, and SVs in the WB samples, diamond v0.8.36 [46] was used to search for Arabidopsis orthologs to the putative causal genes using the Araport 11 Arabidopsis annotation [47] with the following parameters: *--max-target-seqs 1* and *--unal 0*. The list of Arabidopsis genes orthologous to WB candidate genes was loaded into RStudio, and the R/Bioconductor package biomaRt v2.54.1 [48] was used to obtain gene descriptions from Ensembl Plants [49]. The Arabidopsis orthologs and the information about their function were then used to prioritize genes involved in developmental, hormone signaling, or other pathways that could potentially result in the WB phenotype. Variants of interest were verified first by looking at mapped reads for all samples in a genome browser to verify that the genetic variants were truly genotypically distinct to the WB sample. Then, polymerase chain reaction (PCR) was used to validate the variant in all samples. The amplified products were Sanger sequenced to verify that the variant called was accurate in both location and genotype.

## Results

### WB shoot phenotypes

**Fig. 1:**
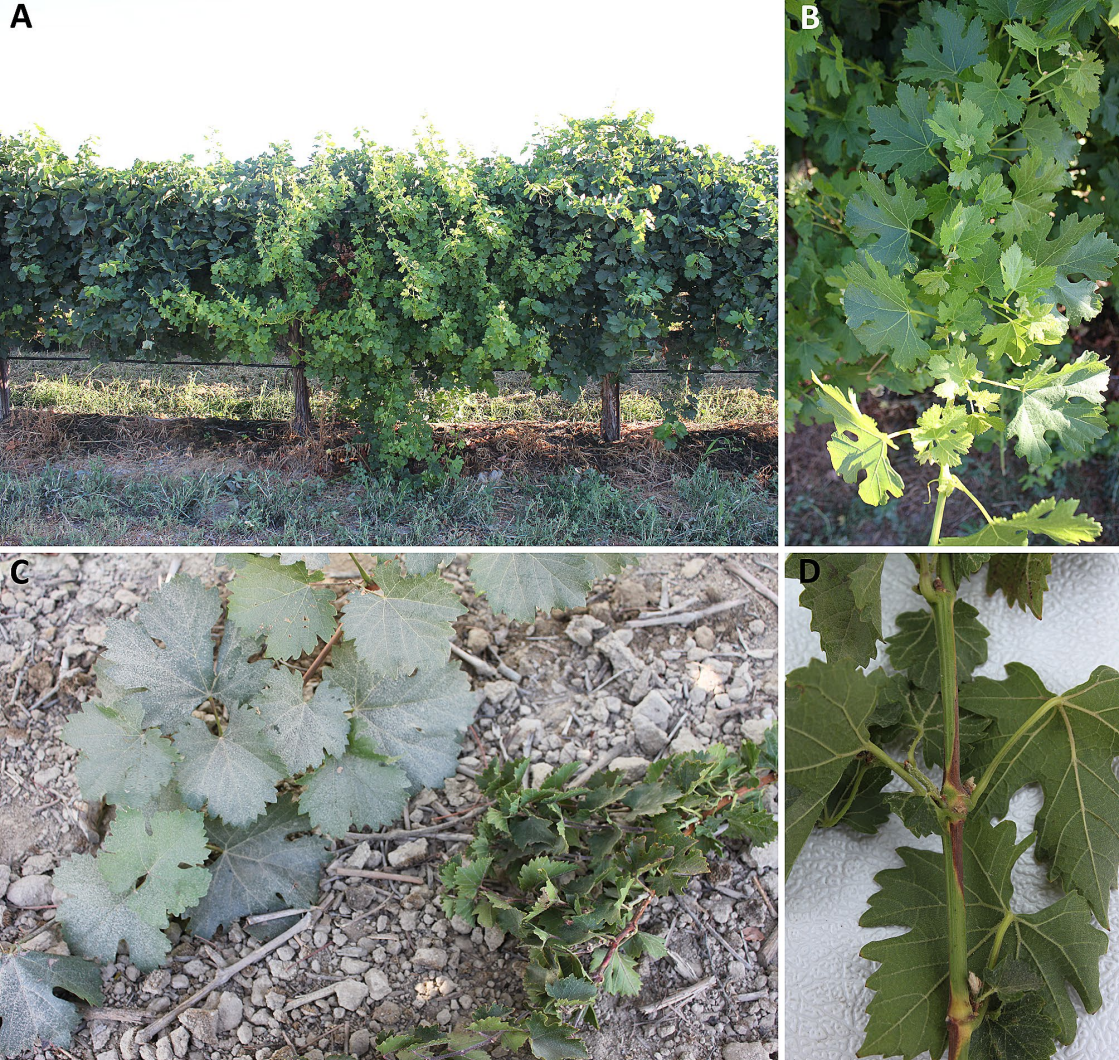
Photos of wild type and Witch’s Broom shoots from a commercial vineyard. **(A)** Photos of Merlot WB and WT on one grapevine plant. WB shoots are the light green shoots in the center of the image, while WT shoots are the darker green shoots on either side of the WB shoots. Merlot WB shoots display prolific growth in comparison to their WT counterparts. **(B)** An up-close photo of Merlot WB shoot, with light green leaves and shortened internodes. **(C)** A side-by-side photo of Dakapo WT (left) and Dakapo WB (right) shoots from different plants. Dakapo WB shoots have shortened internodes and more prolific foliage than their WT counterparts. **(D)** An up-close photo of a Dakapo WB shoot, showing a significantly shortened internode

The WB bud sport arises spontaneously in many varieties of grapevine [6]. We characterized two independent cases of WB that occurred at a commercial vineyard in Madera, CA. The first case is a WB mutant of a Merlot grapevine, observed as one arm (the eastern) on a vine in a commercial vineyard block. The adjacent western arm on the same plant is WT. This allowed a direct comparison of WB and WT tissues from the same plant. The second case characterized was in the Dakapo variety and is a WB vine that was identified as a whole plant mutation. As a result, no WT shoots were present on the Dakapo WB plant, so separate, unaffected Dakapo vines from the same propagation batch were used as the WT comparison. In both cases, the bud sport is characterized by vigorous vegetative growth with shortened internodes (Fig. 1). Both cases of WB also appear to have issues rooting, with Dakapo WB cuttings rooting less frequently than Dakapo WT cuttings, and Merlot WB cuttings being entirely unable to root (P. Cousins, unpublished observations). Merlot WB shoots have light green leaves strikingly distinct from WT shoots (Fig. 1A), while Dakapo WB leaves are similar in color to WT shoots (Fig. 1C).

**Fig. 2:**
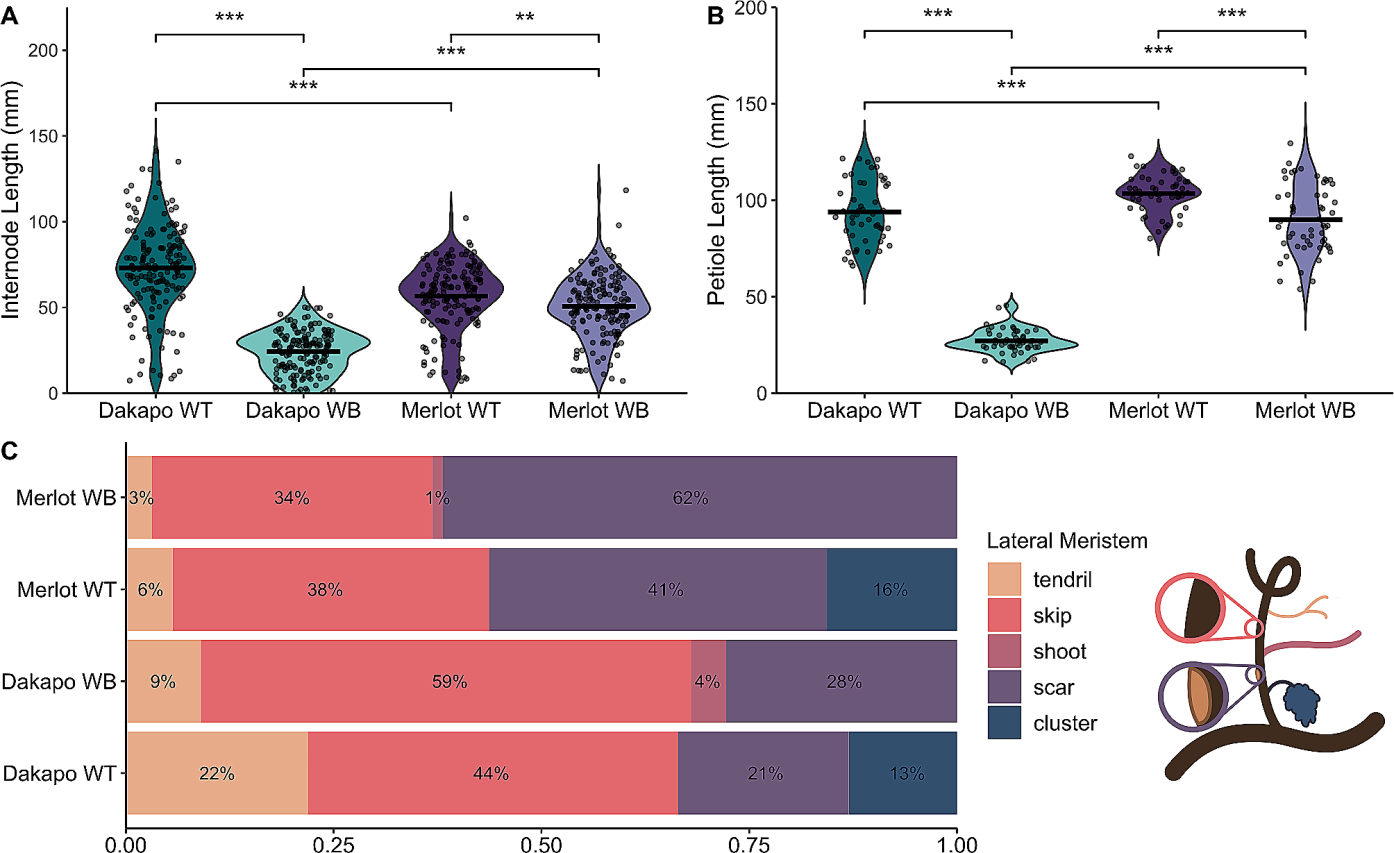
Differences in shoot phenotypes between wild type and Witch’s Broom samples in Dakapo and Merlot varieties of grapevine. **(A)** A comparison of average internode length and **(B)** petiole length between sample types, collected from 10 shoots each. Mean values were represented by a black line for each sample. Dakapo WB and Merlot WB both have significantly smaller internodes (*P* < 0.001*** and *P* < 0.01**, respectively) and petioles (*P* < 0.001*** for both cases) in comparison to WT plants of the same variety. The WT samples of the two varieties differ as well, with Dakapo WT having longer internodes but shorter petioles than Merlot WT (*P* < 0.001*** for both). Dakapo WB also has significantly smaller internodes and petioles compared to Merlot WB (*P* < 0.001*** for both). **(C)** The percentage of nodes with specific lateral meristem outcomes, collected from 144–160 lateral meristems for each sample. The diagram to the right of the legend shows each of the lateral meristem outcomes both in the color and order they appear on the legend

Comparison of multiple shoot traits between the WT and WB plants revealed large differences in phenotypes between the two. Both Dakapo and Merlot WB shoots have internodes significantly shorter than their WT counterparts (t = -21.86, df = 230.76, *P* < 0.001 for Dakapo; t = -2.93, df = 317.25, *P* = 0.003 for Merlot) (Fig. 2A). The petioles were also smaller in WB plants than WT (t = -27.72, df = 32.91, *P* < 0.001 for Dakapo; t = -5.01, df = 87.44, *P* < 0.001 for Merlot) (Fig. 2B). Our phenotyping also revealed that the Dakapo WB phenotype seems to be more severe than the Merlot WB phenotype. The Dakapo WB internodes are significantly shorter than those of Merlot WB (t = -15.54, df = 281.57, *P* < 0.001), despite Dakapo WT internodes being longer than Merlot WT internodes (t = 6.58, df = 294.07, *P* < 0.001) (Fig. 2A). In addition, the Dakapo WB petioles are also significantly shorter than their Merlot WB counterparts (t = -25.19, df = 69.41, *P* < 0.001) (Fig. 2B).

**Fig. 3:**
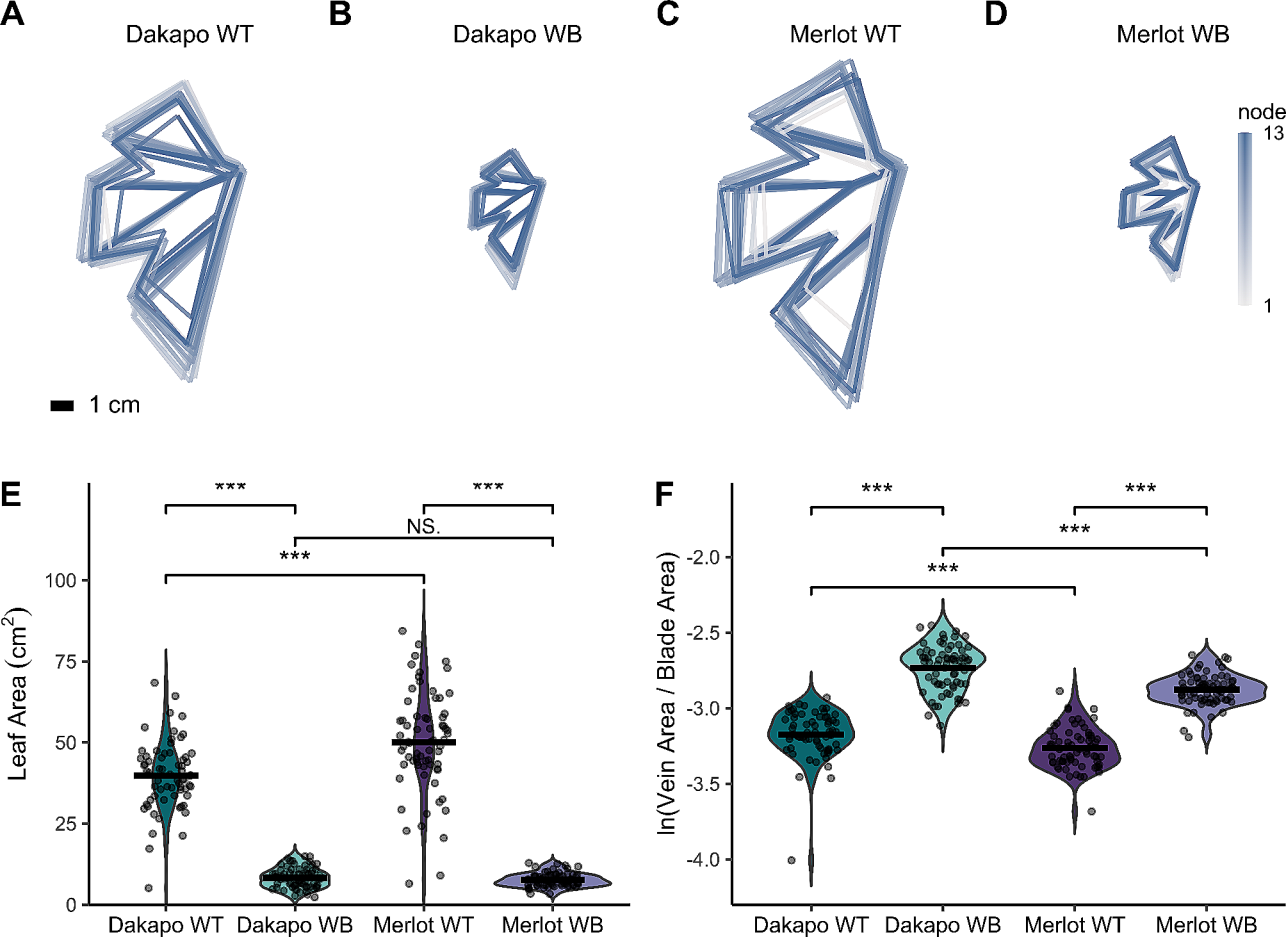
Comparing leaf area and the natural log of the ratio of vein-to-blade area between wild type and Witch’s Broom samples in Dakapo and Merlot varieties of grapevine. **(A)** Dakapo WT, **(B)** Dakapo WB, **(C)** Merlot WT, and **(D)** Merlot WB composite leaves generated using leaf landmarks to model leaf shapes for leaves collected across 13 nodes. Composite leaves are colored based on node, from gray (node 1 from the shoot tip) to dark blue (node 13). All samples are to the same scale, and a 1 cm scale bar is provided in the bottom left corner of **(A)**. **(E)** A comparison of leaf area (cm^2^), as calculated using the shoelace algorithm originally described by Meister (1769) and used in Chitwood et al. (2020) to calculate leaf area in grapevine, with leaf landmark data. Mean leaf area (cm^2^) is represented by a black line for each sample. Dakapo WB and Merlot WB both have significantly smaller leaves (*P* < 0.001*** for both cases) in comparison to WT plants of the same variety. Merlot WT leaves were larger than Dakapo WT leaves (*P* < 0.001***), however leaf area did not differ between the two WB cases (*P* = 0.16). **(F)** A comparison of the natural log of the ratio of vein-to-blade area, an allometric indicator of leaf size that is typically more sensitive to leaf size changes than leaf area alone. Mean ln (vein-to-blade ratio) is represented by a black line for each sample. Dakapo WB and Merlot WB both have significantly higher vein-to-blade ratios (*P* < 0.001*** for both cases) in comparison to WT plants of the same variety. Dakapo WT leaves have a higher vein-to-blade ratio than Merlot WT leaves (*P* < 0.001***). Dakapo WB leaves have a higher vein-to-blade ratio than Merlot WB leaves (*P* < 0.001***) as well

Initial measurements of leaf width and length demonstrated that Dakapo and Merlot WB leaves are significantly shorter and narrower than their WT counterparts when compared at the same node (*P* < 0.05 for width and length at node 4 for Dakapo; *P* < 0.05 for both width and length, for nodes 5–9 for both Dakapo and Merlot cases) (Additional file 1: Fig. S1). While initial data collected in 2021 showed that the Dakapo and Merlot WB leaves were typically shorter and narrower than their WT counterparts (Additional file 1: Fig. S1), the actual change in leaf area and leaf shape was unknown. Leaves collected and landmarked from all samples in 2022 demonstrated that WB leaf areas were significantly smaller overall than their WT counterparts (t = 23.49, df = 76.98, *P* < 0.001 for Dakapo; t = 22.41, df = 70.42, *P* < 0.001 for Merlot) (Fig. 3A-E). To further understand how WB leaf development may differ from typical grapevine leaf development, we calculated the allometric ratio of vein area to blade area. As leaves expand, the blades of leaves expand at a greater rate than the veins [50]. As a result, larger leaves typically have lower vein-to-blade ratios. In addition, the ratio of vein-to-blade area is typically more responsive to subtle changes in leaf shape and development than area alone [14]. As expected, given their small leaves, both Dakapo WB and Merlot WB have significantly higher vein-to-blade ratios in comparison to WT plants of the same variety (t = -16.67, df = 133.14, *P* < 0.001 for Dakapo; t = -19.08, df = 127.55, *P* < 0.001 for Merlot) (Fig. 3F). Dakapo WB leaves also have a higher vein-to-blade ratio than Merlot WB leaves (t = 6.53, df = 120.44, *P* < 0.001) (Fig. 3F). This is likely due to very subtle differences in leaf development between the two WB samples that are not captured by comparing leaf area alone, such as differences in vasculature development between the two.

**Fig. 4:**
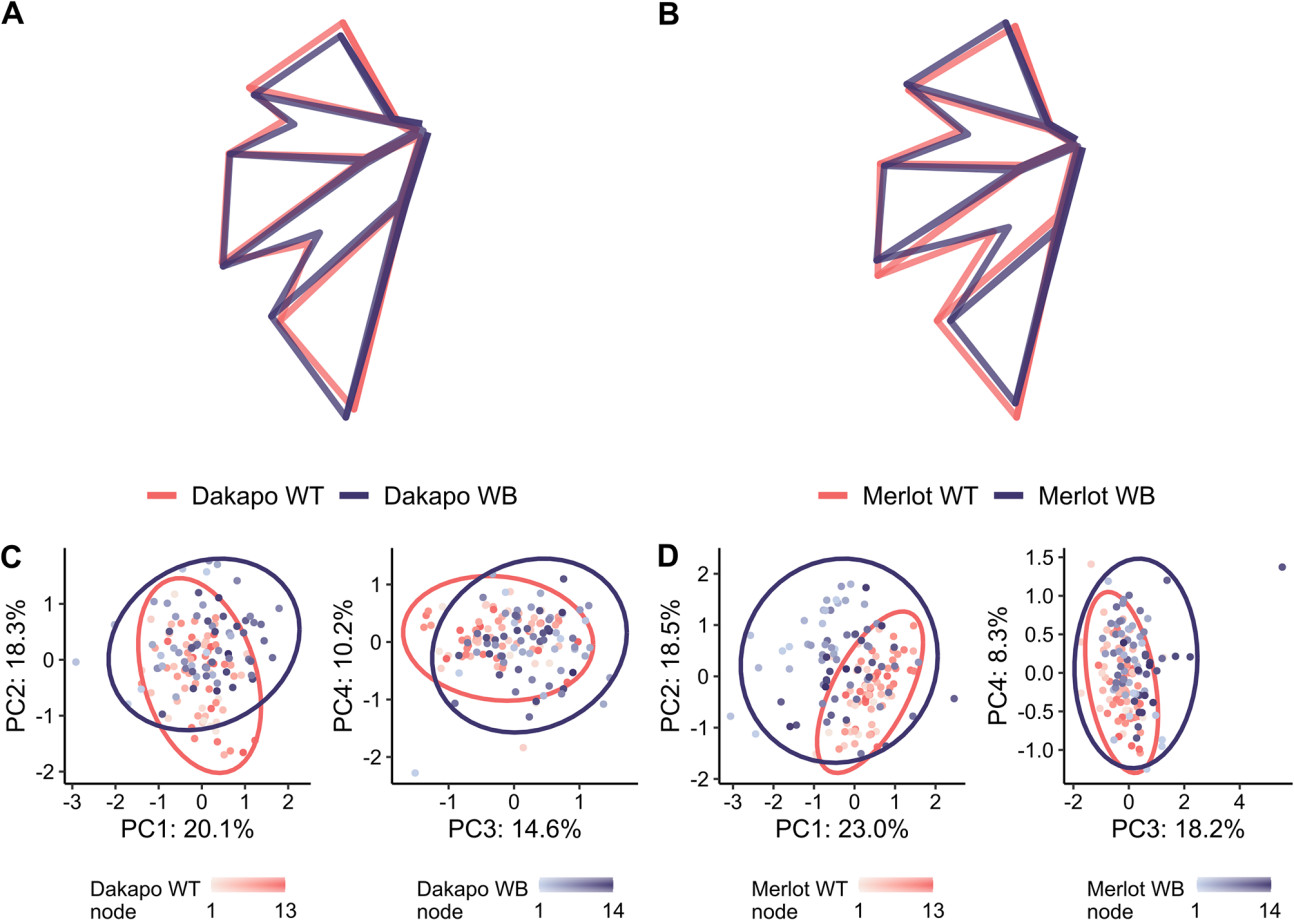
Mean leaf shapes rotated and scaled identically for **(A)** Dakapo WT and Dakapo WB, as well as for **(B)** Merlot WT and Merlot WB. **(C and D)** Principal component analysis (PCA) of all leaf shapes, with WT colored in salmon and WB colored in purple, for **(C)** Dakapo and **(D)** Merlot. The node position of the leaves is also shown by shade, with the lightest shade being node 1 (from the shoot tip) and the darkest shade being node 13–14, depending on the sample

Analyzing the leaf landmark data utilizing a Procrustes analysis and a principal components analysis (PCA) revealed that WB leaves also differ in their shape when compared to their WT counterparts (H = 13.26, *P* < 0.001 for Dakapo; H = 14.07, *P* < 0.001 for Merlot) (Fig. 4). Eigenleaves from the PCA comparing leaf shape between scaled WT and WB leaves (Additional file 2: Fig. S2 and Fig. S3) revealed the shape features that each PC reflected. The leaf shape variance between WT and WB in both Merlot and Dakapo appears to be due to similar phenotypic changes in the WB leaves. For both varieties, PC2 reflects variance in the depth of the distal sinus, which is deeper in WB samples. WB leaves in both varieties also seem to have a wider petiolar sinus, which is reflected by PC3 in Dakapo and PC4 in Merlot. Additionally, WB plants in both varieties appear to have narrower upper lateral lobes, which is explained by PC4 in Dakapo and PC1 in Merlot (Fig. 4). Despite these similarities in how WB leaves differ from WT in the two varieties, WB also appears to impact leaf shape somewhat differently in the two varieties. Dakapo WB leaves appear to have narrower distal sinuses than their WT counterparts, as described by PC1 (Fig. 4A, C). Meanwhile, Merlot WB leaves appear to have shorter midveins relative to the rest of leaf features, as explained by PC3 (Fig. 4B, D). These two features appear to be specific to WB bud sports of the particular variety.

We also characterized the fates of lateral meristems of the WB bud sports to understand the developmental outcomes of the WB buds. Lateral meristem fates were characterized by the organ or structure that had developed at the nodes, which were either: (a) tendrils, (b) skips (nodes where no lateral meristem was present), (c) shoots, (d) scars (nodes where a meristem had formed, but no structure was present when phenotyped), or (e) clusters/fruit. These observations revealed that no clusters were developing in the WB shoots. In addition, the WB shoots developed new lateral shoots at 1–4% of nodes, while their WT counterparts did not develop these new lateral shoots at any nodes (Fig. 2C). New grapevine shoots arise from axillary buds, and lateral shoots typically do not develop. It is possible that the incidence of lateral shoots on the WB bud sports may be due to the mutation directly. Both the presence of the lateral shoots and absence of clusters support that the WB bud sports seem to involve a shift towards vegetative growth and away from reproductive growth. Many of the WB lateral meristems failed to develop properly, with 87% of Dakapo WB buds and 96% of Merlot WB buds failing to develop into tendrils, clusters, or shoots, compared to 65% and 79% in their respective WT counterparts. The higher incidence of skips in Dakapo WB (59%), in comparison to Dakapo WT (44%), contributes directly to the lack of tendrils and clusters observed. Dakapo WB having more skips present is somewhat unexpected, as grapevines are expected to generally show a phyllotaxy of two successive nodes with a lateral meristem followed by one node without. As a result, we generally expect to see 1/3 of the nodes studied to be skips. It is possible that the WB mutation in Dakapo causes an unusual phyllotaxy and thus more skips to be present. However, the Merlot WB shoots have about the expected number of skips present (34%). While the characterization of lateral meristem fate demonstrated dominance of vegetative growth in both instances of WB, it also revealed that they may have distinct issues when it comes to lateral meristem development.

### Organization and development of WB buds

**Fig. 5:**
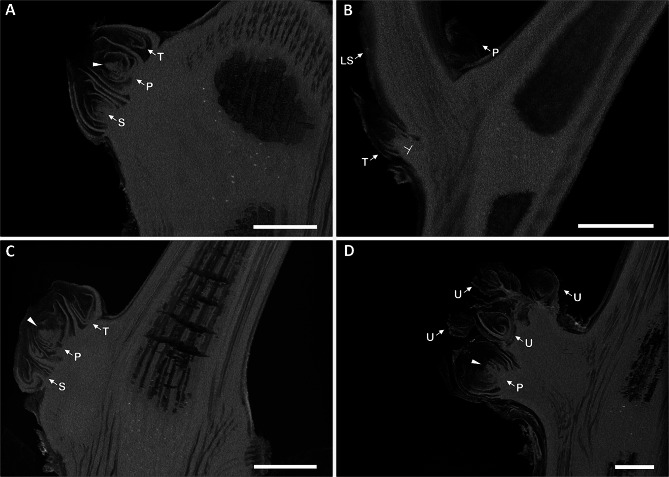
Representative CT scans of buds from **(A)** Dakapo WT, **(B)** Dakapo WB, **(C)** Merlot WT, and **(D)** Merlot WB samples. Primary (P), secondary (S), and tertiary (T) bud primordia are indicated if present in the image. The inflorescence primordia are indicated by the solid triangle in the **(A)** Dakapo WT, **(C)** Merlot WT, and **(D)** Merlot WB samples. Only one inflorescence primordium is present in the images, although multiple were seen for both WT samples. The Merlot WB sample shown **(D)** is the only Merlot WB sample scanned with a potential inflorescence primordium present, although the inflorescence primordium seen appears to be deformed due to the edges being smoother than those seen in WT samples (**A and C)**. The lateral shoot stem (LS) is indicated in **(B)** Dakapo WB. The bud primordia in the Merlot WB sample shown are challenging to accurately label, aside from the more-developed primary primordia (P), so we have labeled the additional bud primordia as uncharacterized primordia (U) in **(D)** Merlot WB, which are indicated as well. Scale bar = 1 mm

To investigate the developmental origin and timing of the defects seen across the WB shoots, particularly in lateral meristem fates, dormant winter buds were imaged to identify changes in bud organization. To do so, we imaged dissected grapevine buds with a dissecting scope and whole buds with CT scans. Grapevine dormant winter buds are typically composed of three bud primordia, characterized as primary, secondary, and tertiary, from most developed to youngest respectively. The bud primordia typically house leaf, tendril, and inflorescence primordia [3]. The WT buds for both Dakapo and Merlot varieties had nearly identical organization and structures. The buds and primordia were each at a 45° angle from the stem. All WT buds had three bud primordia in each of the buds as expected. CT scans showed that all WT buds had inflorescence primordia present, with 80% of WT buds having two or more inflorescence primordia present in their primary bud primordia alone. None of the WT buds appeared to have any organizational defects in the buds, with all primordia appearing to be healthy and properly arranged (Fig. 5A, C, Additional file 3: Fig. S4 A, C, and Additional file 4: Fig. S5 A, C).

In contrast, the WB buds contained multiple organizational defects. Upon examination, about half of the Dakapo WB buds had an initiated lateral shoot stem extended out of them, about 1 cm long (Additional file 3: Fig. S4B and Additional file 5: Fig. S6). CT scans revealed that this stem appears to be vascular tissue pushing through the bud, disrupting the typical organization (Fig. 5B and Additional file 4: Fig. S5B). The vascular tissue expanding through the buds sometimes contained a shoot apex on the tip, suggesting that these shoots can produce leaves and other lateral organs. Half of the buds contained an additional change in overall architecture, with the tertiary primordia being perpendicular to the stem (Fig. 5B). Many of the primordia present in the Dakapo WB buds appeared to be smaller than those in the other samples. Notably, three of four Dakapo WB buds had only one inflorescence primordia present, but the inflorescence primordia appeared deformed in two of the buds scanned.

The Merlot WB buds had drastically different organization from WT buds as well, with the buds containing between 4 and 8 bud primordia (Fig. 5D, Additional file 3: Fig. S4D, and Additional file 4: Fig. S5D), in contrast to the 3 consistently found in wild type samples (Fig. 5A, C, Additional file 3: Fig. S4 A, C, and Additional file 4: Fig. S5 A, C). Similarly to the Dakapo WB samples, two out of five of the Merlot WB buds had tertiary primordia nearly perpendicular to the stem. In addition, all but one of the Merlot WB buds had no inflorescence primordia. The inflorescence primordium potentially present in the single sample was difficult to confidently identify as such however since it lacks the lobes typically seen in developing inflorescence primordia (Fig. 5D). As a result, even if this structure is truly an inflorescence primordium, it is extremely deformed. However, none of the Merlot WB buds displayed the vascular tissue expansion seen in the Dakapo WB samples.

Overall, the Dakapo and Merlot WB buds contained phenotypes vastly different from WT and even one another. The WB samples displayed extensive defects in bud organization and the quantity of inflorescences produced indicating that the WB defects manifested early in bud development. This investigation into the buds of the WB bud sports provided insight into the defects we identified across the shoots of the bud sports. Not only are the shoots failing to develop properly, but the defects are pervasive in the buds and potentially their internal structures, as well.

### Genetic variation in WB Bud sports

To investigate the genetic basis of the WB bud sport, we sequenced DNA from both Dakapo and Merlot WB and WT using both Illumina 150 bp paired-end sequencing and Oxford Nanopore long-read sequencing. After trimming and filtering, the sequencing coverage of the Illumina reads was between 49-57X and the sequencing coverage of the Oxford Nanopore reads was between 18-31X (see Additional file 6: Table S1 for full sequencing statistics). The read length N50 for the trimmed Oxford Nanopore reads was between 12,890 − 14,486 bp for the samples. High quality reads were used for mapping to the reference genome and calling variants in each of the samples. For all samples, over 98.2% of Illumina reads and over 99.9% of the Oxford Nanopore reads mapped to the grapevine 12X.v2 reference genome [26].

SNVs and INDELs were called against the 12X.v2 grapevine reference genome [26] using Illumina sequencing data. Each sample had between 7.9 and 8.2 million SNVs/INDELs and high heterozygosity (67.96–71.22%). Most SNVs and INDELs were present in both WT and WB samples of the same variety (94.81–94.97%), however between 409,588 − 418,818 SNVs/INDELs were entirely novel when compared within-variety. A majority of SNVs and INDELs were either intergenic or not expected to have an impact on gene function (Additional file 7: Table S2). Of the SNVs and INDELs called in the WB samples, 6,296-6,450 were predicted to have high impact on gene function. Between 597 and 613 genes impacted by SNVs or INDELs predicted to have a high impact were genotypically distinct in WB bud sports, and these genes were kept as possible causal candidates for the bud sport (Table 1).

**Table 1:** Genetic variants identified in samples when called against the 12X.v2 grapevine reference genome assembly, including SNVs/INDELs and SVs. Novel and genotypically distinct variants were identified by comparing variants intra-variety

	Dakapo		Merlot	
	WT	WB	WT	WB
SNVs and INDELs				
Total SNVs/INDELs	7,912,797	7,925,441	8,148,571	8,163,074
Genotypically distinct SNVs/ INDELs^a^	497,531	493,394	495,782	503,973
Novel SNVs/INDELs^b^	410,608	411,425	409,588	418,818
High impact SNVs/INDELs	14,013	13,962	14,032	14,027
Genes impacted by high impact SNVs/INDELs^c^	6,310	6,296	6,450	6,445
Genes impacted by genotypically distinct high impact SNVs/INDELs	611	613	591	597
**SVs**				
Total SVs	52,119	53,089	54,775	53,912
Genotypically distinct SVs^a^	578	635	691	540
Novel SVs^b^	157	223	224	102
Genes impacted by SVs	15,044	15,134	15,706	15,596
Genes impacted by genotypically distinct SVs	135	136	150	134

^a^ Variants genotypically distinct from the sample of the same genotype include entirely novel SNVs, as well as SNVs that have different genotypes when compared intra-variety

^b^ Novel variants are variants completely absent in the sample of the same variety

^c^ High impact SNVs include frameshift deletion or insertion, stop gain/loss, and splicing

Structural variants were called against the 12X.v2 grapevine reference genome [26] using long-read sequencing data. Each sample had between 52 and 55,000 SVs. Deletions were the most common type of SV and accounted for over half of the SVs called. Insertions were the next most common type of SV and accounted for about 47% of total SVs. Inversions, transversions, and duplications were extremely rare, and collectively only accounted for between 1.27 and 1.66% of all SVs called (Additional file 8: Table S3). Entirely novel SVs (when compared within variety) were rare as well, with only between 102 and 224 identified within the samples. Only 635 and 540 SVs were genotypically distinct in Dakapo WB and Merlot WB, respectively. About 15,000 genes had SVs within them for each sample individually. Of the genes containing SVs, 136 and 134 were impacted by genotypically distinct SVs for Dakapo WB and Merlot WB, respectively (Table 1).

We identified 974 and 968 high impact SNVs, INDELs, and SVs genotypically distinct in Dakapo WB and Merlot WB respectively that are all potential candidates for the WB bud sport in their respective genotype (Additional file 9: Table S4). We looked at the gene function for 577 and 561 genes only impacted by high impact mutations in WB samples in Dakapo WB and Merlot WB, respectively. The two WB samples shared 164 genes impacted by high impact variants. All genes in common between the two WB samples were weak candidates with either gene functions unrelated to the WB phenotype or were unsupported by the genome browser and/or PCR validation. As a result, we investigated the potential biological impact and validity of the 974 high impact variants in Dakapo WB and the 968 high impact variants in Merlot WB, separately. To narrow down this list of potential candidates for both cases of WB, we looked at the function of the genes impacted by these variants and further investigated genes involved in development, growth, or hormone signaling. Most genes impacted by high impact variants were involved in unrelated processes or were of unknown function, however 14 variants in Dakapo WB and 23 variants in Merlot WB were identified has having a high impact on genes involved in development, growth, or hormone signaling. We looked at WT and WB reads mapping at the loci of these variants as an initial pass to ensure that they were accurately genotyped, and only one high impact variant for both Dakapo WB and Merlot WB seemed to truly be genetically distinct to the WB case of interest. PCR validation of these two variants demonstrated that the Dakapo WB variant of interest was present in a heterozygous state in both Dakapo WT and WB and therefore likely not a strong genetic candidate for the Dakapo WB bud sport. However, PCR validation and Sanger sequencing demonstrated that the Merlot WB variant was present in Merlot WB only and was entirely absent in Merlot WT (see Additional file 10 for methods; Additional files 11–12: Fig. S7 and Fig. S8). The PCR-validated variant in Merlot WB is a 3.6 kbp insertion in the intron of GSVIVG01008260001 (VCost.v3 annotation gene ID: Vitvi17g00344 [26], CRIBI V1 annotation gene ID: VIT_17s0000g03960 [51]), an ortholog of Arabidopsis STOMATAL CYTOKINESIS-DEFECTIVE 1 (Additional file 13: Fig. S9). This variant is heterozygous in Merlot WB and completely absent in Merlot WT samples. A BLASTN search against the 12X.v2 grapevine reference genome assembly [26] showed that this sequence showed significant similarity to 2,736 sequences within the genome, spread across all 19 chromosomes. The 3.6 kbp insert sequence also contains seven transposable element sequences that account for 98.5% of the sequence, including four *Gypsy* long terminal repeat (LTR) retrotransposons, two uncharacterized LTR retrotransposons, and one *Mutator* terminal inverted repeats (TIR) retrotransposon (see Additional file 10 for methods). Of these, one *Gypsy* LTR and one uncharacterized LTR appear twice in the insert sequence adjacent to one-another. We propose that this genetic variant may be the causal mutation for the WB bud sport in the Merlot WB case investigated.

## Discussion

### Developmental defects in the WB bud sport

Our phenotypic measurements of the Dakapo and Merlot WB bud sports revealed new aspects of the WB phenotype that had previously been unknown. The most striking finding being how different the two instances of WB studied are from one another, with the Dakapo WB shoots having much smaller features in comparison the Merlot WB (Figs. 2A and B and 3E). Analysis of lateral meristem fate, leaf shape, and dormant buds further enforced how distinct the two instances of WB are (Figs. 2C and 4, and Fig. 5B, D). However, both WB cases were significantly smaller than their wild type counterparts in every trait measured. The WB phenotype also seems to include development defects that have not been previously identified, such as subtle changes in leaf shape in both varieties (Fig. 4). The phenotypic measurements across the shoots of the WB bud sports show that not only are they smaller than their WT counterparts, but they also have defects in regulating overall shoot and leaf development. Our leaf size and shape data both seem to support that the WB leaves specifically seem to have very distinct developmental trajectories, with (a) WB leaf areas not following the negative quadratic trend we expect to see as leaves age (Additional file 14: Fig. S9) and (b) WB leaves across the shoots having juvenile characteristics, such as deeper sinuses [11] (Fig. 4). Identifying lateral meristem fates and analyzing internal bud morphologies also clarified developmental defects within the two instances of WB. These results suggested that the WB phenotype may be largely influenced by issues early on in meristem development, leading to a diverse array of developmental defects.

### Investigating the genetic basis of the WB bud sport

Given the phenotypic differences between Dakapo WB and Merlot WB, it is possible that there are multiple genetic means of causing what is colloquially termed a “Witch’s Broom bud sport”. Mutational Witch’s Brooms are poorly described in angiosperms, although they are described from conifers [10], leading to few likely candidate genes in which mutations may drive the WB phenotype. Due to the large differences in phenotype between the two varieties, as well as none of the shared genes impacted by variants being good candidates for the bud sport, we propose that two different genetic variants cause the WB bud sport in the Dakapo and Merlot cases we investigated.

In Merlot, we identified a putative candidate gene for WB: GSVIVG01008260001. It is highly expressed in most tissue types, including buds, leaves, inflorescences, and roots [52], making it a promising candidate for a mutation with pleiotropic effects. GSVIVG01008260001 is orthologous to the gene AT1G49040 in Arabidopsis, which encodes STOMATAL CYTOKINESIS-DEFECTIVE 1 (AtSCD1). AtSCD1 is involved in the cytokinesis of guard mother cells and other leaf epidermal cells. However, AtSCD1 also appears to play a role in overall plant growth and development. In Arabidopsis, *scd1* mutants are smaller than WT plants, have reduced leaf expansion, and defects in flower morphology. The floral buds in *scd1* are smaller than WT due to early abortion in development and are highly branched as well [53]. The phenotype of the *scd1* floral buds is similar to the WB phenotype of Merlot WB buds, which are smaller than WT and also highly branching (Fig. 5D). The dwarfness and small leaves of *scd1* also match what we see in Merlot WB shoots. The abundant similarities between *scd1* mutants in Arabidopsis and the Merlot WB bud sport make GSVIVG01008260001 a strong candidate for one casual gene of the WB bud sport. While the 3.6 kbp insertion in Merlot WB is heterozygous and there is no explicit support for this mutation being a dominant loss-of-function mutation, both Merlot WT and Merlot WB contain shared heterozygous high impact variants (Additional file 15: Table S5), including a frameshift deletion and a splicing variant that both would be expected to impede the function of GSVIVG01008260001. It is possible that one or more of these heterozygous variants shared by Merlot WT and WB cause one copy of the gene to be nonfunctional and that the Merlot WB-specific 3.6 kbp insertion causes the other copy to be nonfunctional as well, resulting in Merlot WB having no functional copy of GSVIVG01008260001. The insert sequence within GSVIVG01008260001 being almost entirely annotated as TE sequence also provides a clear possible explanation for how this bud sport could arise spontaneously since the TE sequence within the insertion may have led the insertion within this gene. Additionally, no other genes overlapping with SNVs or SVs unique to Merlot WB appear to be strong candidates. Most other genes identified as uniquely impacted by variants in Merlot WB do not appear to be involved in plant growth and development and/or are not truly genetically distinct in Merlot WB. Between the genetic evidence in the Merlot WB grapevine plants and phenotypic similarity to the Arabidopsis ortholog [53], we propose GSVIVG01008260001 as a candidate causal gene for the WB bud sport in grapevine.

While we were able to identify a strong candidate in Merlot WB, no strong candidates were identified in Dakapo WB. There are a few complicating factors that contributed to the difficulty of identifying a causal WB candidate in our Dakapo WB sample. For one, grapevine is highly heterozygous, which made it challenging to both accurately call and genotype SNVs and SVs within our samples. In addition, genetic chimeric variability, in which one cell layer has distinct genetic variants in comparison to the other cell layer, has repeatedly been identified in grapevine [54, 55]. The phenotypic manifestation of a chimeric genetic variant depends on the cell layer(s) it is present within [56]. As a result, it is possible that the WB causal variant could be present in both WT and WB sequencing data, but present in distinct cell layer(s) between WT and WB. If the WB causal variant is chimeric in nature, it may not have been identified through our sequencing and variant calling. Finally, it is also possible that the WB bud sport could be the result of an epiallele as well, as was found with the mantled somaclonal variant that arises frequently in oil palm [57].

Ultimately, genetic transformation is necessary to prove the causal gene(s) of the WB bud sport. However, it is likely that an inducible mutant will need to be used to circumvent possible lethality due to issues that the bud sports have with rooting. As a result, the natural instances of the WB bud sport studied here provide invaluable natural mutants for studying whole plant development in grapevines. It is possible that the WB bud sport provides insight into developmental defects and interactions between developmental processes that might otherwise be impossible to study due to the inability of the WB bud sports to properly root and produce seed. Studying other occurrences of WB in the future will provide more insight into grapevine development and clarify the extent of the phenotypic and genetic diversity of “Witch’s Broom bud sports”.

### Somatic mutations in grapevine shoots and clones

Our paired sequencing of WT and WB tissue from two instances of WB in grapevine also provided insight into somatic mutations both between clones and within plants. All samples had relatively similar counts of sample-unique SNVs when compared within variety (Table 1). We found between 349,239 and 349,533 of clone-specific SNVs in Dakapo (Additional file 16: Table S6). This is somewhat lower than what has been found in other studies performing a 1:1 comparison of only two clones of the same variety, as we have done, which ranged from $\sim$ 600k-3.3 million SNVs [58, 59]. This distinction is likely due to differences in methods employed, as these studies compared clone sequencing data to the reference genome of the same variety, while we performed joint variant calling and genotyping against the grapevine reference genome. However, our count is higher than clone-specific SNVs that have been identified when comparing larger populations of grapevine clones with more than two individuals, which ranges from 200 to 30.7k SNVs [58–61]. Studies looking at intra-clonal variation in grapevine have all had different aims and thus different variant calling and filtering approaches, which has likely led to this large range in the number of SNVs identified both between clones and within varieties. Our data also provided insight into intra-organism mutations in grapevine, which have been relatively understudied compared to intra-clonal mutations. Our dataset revealed that the number of somatic mutations within one grapevine plant, when comparing distinct shoots (Merlot WT shoots and Merlot WB shoots), is similar to those found between grapevine clones (Table 1), with between 351,018 and 356,754 shoot-specific SNVs being identified in Merlot (Additional file 16: Table S6). The counts of shoot-specific SNVs in Merlot is higher than the number of intra-organisms SNVs that have been identified in grapevine (3.2-3.7k) [62] and other plant systems (4.9k SNVs in *Zostera marina* and 44-152k SNVs in *Populus trichocarpa*) [63, 64]. This is likely in large part due to the differences in methods used between our study and previous studies due to the differences in the aims of the studies. Given that the main goal of this study was to identify putative causal variants of WB, we did not apply stringent filtering that these previous studies have applied [62–64].

Our long-read sequencing also provided insight into SV somatic mutations, which are relatively understudied in comparison to SNV somatic mutations, especially at the intra-organism level. We identified between 157 and 223 clonal-specific SVs in Dakapo, and between 102 and 224 shoot-specific SVs in Merlot. These findings align with our SNV data and support that the number of intra-organism somatic mutations in grapevine is similar to the number of inter-clone somatic mutations. The actual number of clonal- and shoot-specific SNVs and SVs is likely much lower than what was reported due to sequencing errors, alignment errors, etc. Regardless, these data provide insight into the accumulation of mutations within grapevine and supports the notion that grapevine clonal genetic diversity begins through novel somatic mutation accumulations on grapevine shoots, which are then clonally propagated.

## Conclusion

The WB bud sport provides a natural mutant in which to study developmental defects that might otherwise be impossible to study. Grapevine development is vastly different from that in Arabidopsis, and understanding this process and the genetic pathways involved will be invaluable in not only other perennial crop systems, but also in understanding liana development. However, studying the genes involved in grapevine development is difficult due to both traditional breeding and genetic transformation being relatively challenging and time consuming [65]. Investigating the phenotypic defects and potential genetic basis of the WB bud sport has provided insight in grapevine development from buds to shoots. Future work in WB plants, especially with instances of the bud sport in new varieties and genetic backgrounds, will help deepen our understanding of development in grapevine, as well as other lianas and perennial crops.

### Electronic supplementary material

Below is the link to the electronic supplementary material.

Supplementary Material 1

Supplementary Material 2

Supplementary Material 3

Supplementary Material 4

Supplementary Material 5

Supplementary Material 6

Supplementary Material 7

Supplementary Material 8

Supplementary Material 9

Supplementary Material 10

Supplementary Material 11

Supplementary Material 12

Supplementary Material 13

Supplementary Material 14

Supplementary Material 15

Supplementary Material 16

## Data Availability

The phenotypic data from this study are available on Dryad: 10.5061/dryad.5qfttdzdm. Sequencing data from this study are provided on the NCBI Sequence Read Archive under BioProject PRJNA1020818. The code used for data analysis in this study is available on GitHub: https://github.com/eleanore-ritter/witchs-broom/.
