# Supplementary material for: From buds to shoots: insights into grapevine development from the Witch’s Broom bud sport
Source: BMC Plant Biol. 2024 Apr 16;24:283. doi: 10.1186/s12870-024-04992-y (PMC11020879; doi:10.1186/s12870-024-04992-y)
Supplement: Supplementary file 1 — Supplementary Material 1 [file 12870_2024_4992_MOESM1_ESM.pdf]

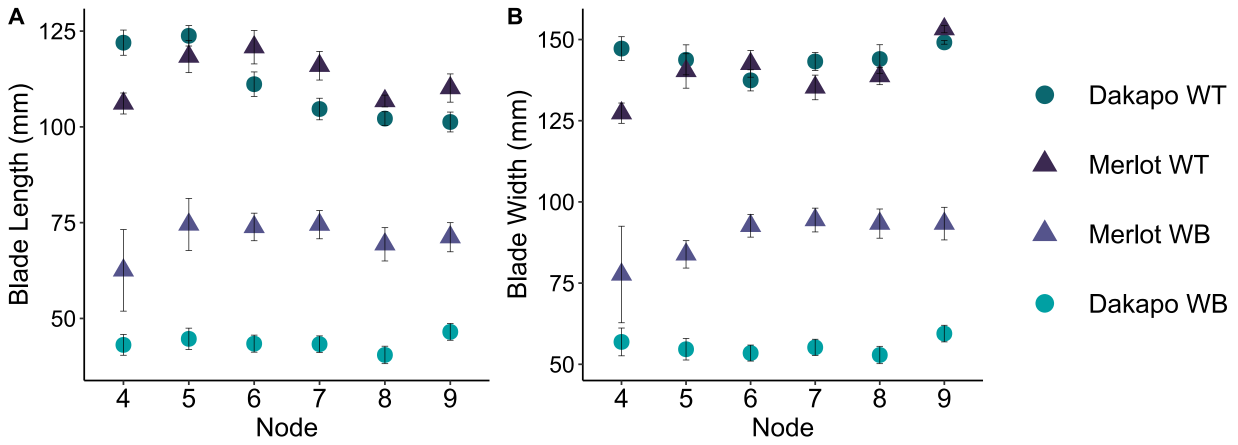

Figure S1. Average leaf **(A)** blade length and **(B)** blade width at distinct nodes on the shoots for each sample, collected from 10 shoots each.
