# Supplementary material for: From buds to shoots: insights into grapevine development from the Witch’s Broom bud sport
Source: BMC Plant Biol. 2024 Apr 16;24:283. doi: 10.1186/s12870-024-04992-y (PMC11020879; doi:10.1186/s12870-024-04992-y)
Supplement: Supplementary file 2 — Supplementary Material 2 [file 12870_2024_4992_MOESM2_ESM.pdf]

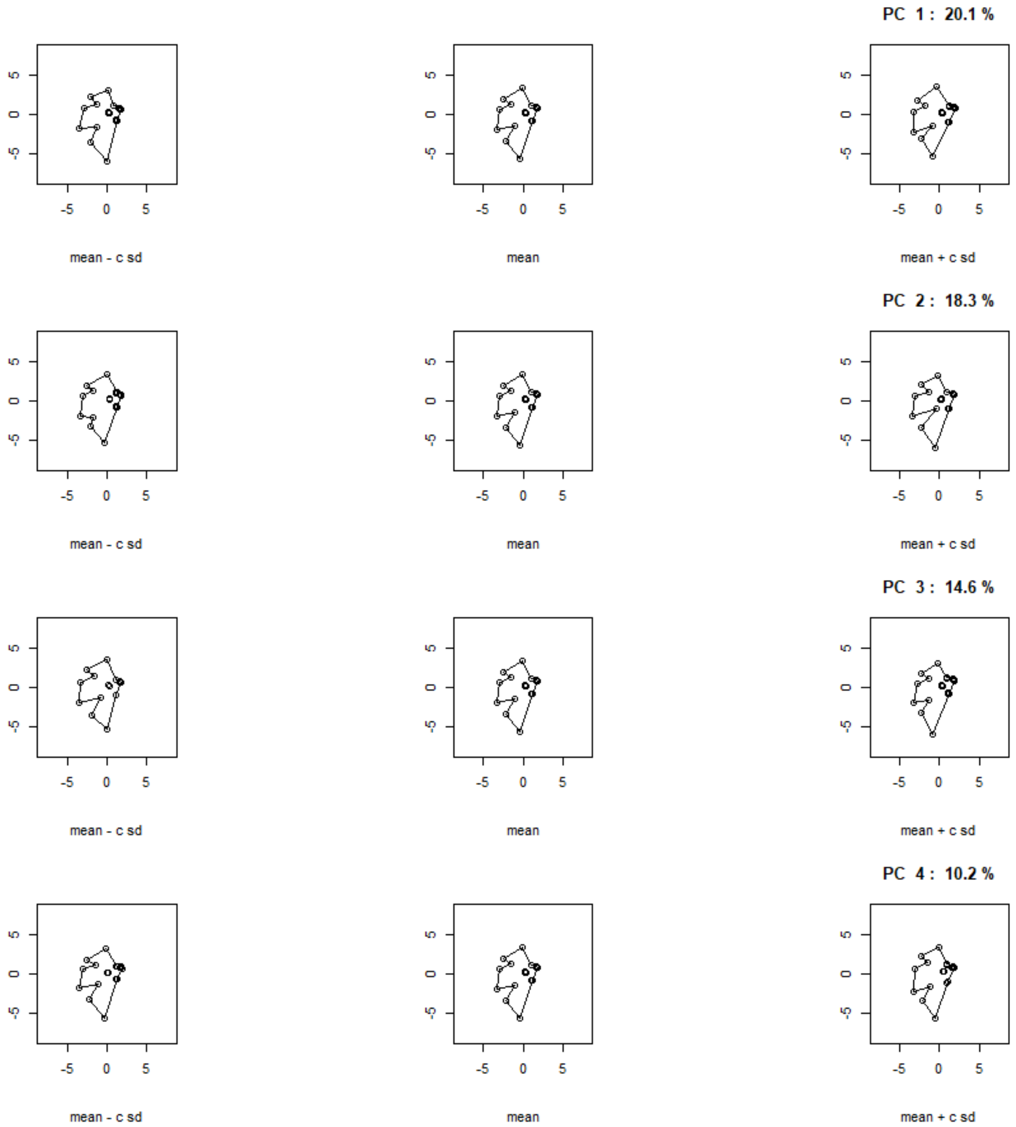

Figure S2. Eigenleaves from the PCA comparing leaf shape between scaled Dakapo WT and Dakapo WB leaves, for PC 1-4.

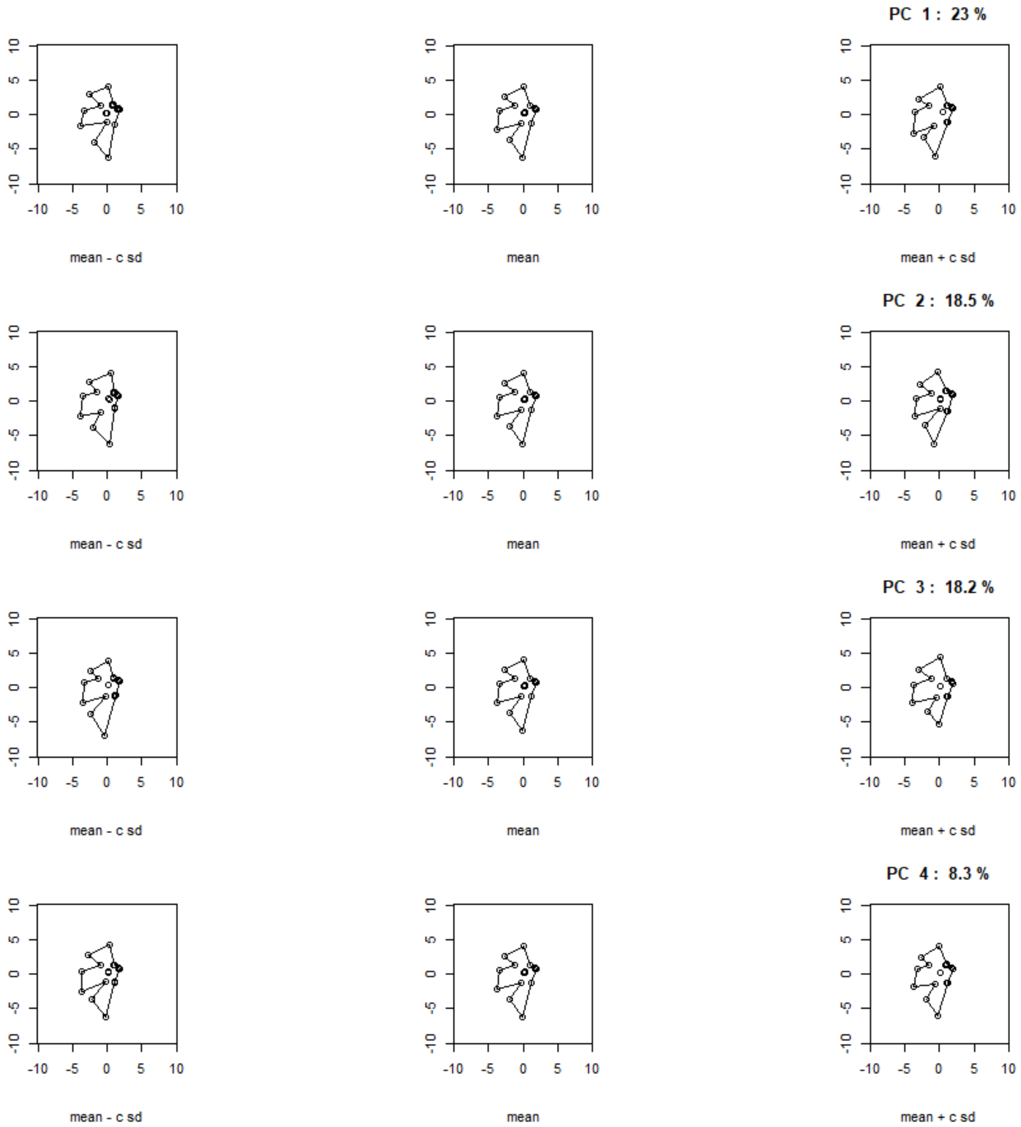

Figure S3. Eigenleaves from the PCA comparing leaf shape between scaled Merlot WT and Merlot WB leaves, for PC 1-4.
