# Supplementary material for: From buds to shoots: insights into grapevine development from the Witch’s Broom bud sport
Source: BMC Plant Biol. 2024 Apr 16;24:283. doi: 10.1186/s12870-024-04992-y (PMC11020879; doi:10.1186/s12870-024-04992-y)
Supplement: Supplementary file 3 — Supplementary Material 3 [file 12870_2024_4992_MOESM3_ESM.pdf]

	Dakapo		Merlot	
	**WT**	**WB**	**WT**	**WB**
Deletions	27,173	27,420	28,495	28,122
Insertions	24,492	24,911	25,677	25,326
Inversions	65	71	67	63
Transversions	662	754	636	575
Duplications	57	58	56	52

Table S3. SV types for all four samples individually, when called against the 12X.v2 grapevine reference genome (Canaguier et al., 2017) using long-read sequencing data.
