# Supplementary material for: From buds to shoots: insights into grapevine development from the Witch’s Broom bud sport
Source: BMC Plant Biol. 2024 Apr 16;24:283. doi: 10.1186/s12870-024-04992-y (PMC11020879; doi:10.1186/s12870-024-04992-y)
Supplement: Supplementary file 4 — Supplementary Material 4 [file 12870_2024_4992_MOESM4_ESM.pdf]

	Dakapo		Merlot	
**Predicted SNP Effects**	**WT**	**WB**	**WT**	**WB**
**downstream**	302954	302701	308529	309713
**intergenic**	4485234	4494808	4559676	4570032
**intronic**	2451458	2454637	2594527	2597389
**ncRNA_exonic**	17	22	14	14
**splicing**	2448	2428	2456	2459
**upstream**	369709	372057	377116	377983
**upstream; downstream**	40420	40459	41191	41112
**UTR3**	68803	68712	70248	70432
**UTR5**	47161	47189	48194	48313
**exonic**				
frameshift	7827	7738	7814	7798
nonframeshift	3446	3443	3515	3532
nonsynonymous	115910	115683	117985	117927
stop gain	3382	3404	3391	3389
stop loss	479	485	495	499
synonymous	90373	89946	92738	92715
unknown	148	145	147	162

Table S2. Number of SNPs with predicted SNP effects for all four samples individually, when called against the 12X.v2 grapevine reference genome (Canaguier et al., 2017) using Illumina sequencing data.
