# Supplementary material for: From buds to shoots: insights into grapevine development from the Witch’s Broom bud sport
Source: BMC Plant Biol. 2024 Apr 16;24:283. doi: 10.1186/s12870-024-04992-y (PMC11020879; doi:10.1186/s12870-024-04992-y)
Supplement: Supplementary file 6 — Supplementary Material 6 [file 12870_2024_4992_MOESM6_ESM.pdf]

## ADDITIONAL METHODS

### Variant validation

In order to validate the insertion in GSVIVG01008260001, we first performed PCR amplification using primers for the wild-type sequence around the 3.6 kbp insertion. These primers were ~27 bp upstream of the start of the insertion and ~267 bp downstream of the insertion, and their sequences were:

**VvSCD1-Forward:** AGCACAATGAAGGAAAACGTGA

**VvSCD1-Reverse:** CTCAACCGGTTACCAAGACGCG

The expected size of the wild-type DNA fragment amplified by these primers was expected to be ~294 bp in length, while the WB DNA fragment was expected to contain the full insertion sequence and be ~3901 bp in length. PCR was performed using the Q5® High-Fidelity DNA Polymerase (M0491; New England Biolabs) and following the manufacturer's protocol for 25 µL reactions, only modifying the concentration of primers by adding 0.5 µL of 10 µM primers. PCR was performed using both MWT and MWB DNA from ONT sequencing in separate reactions. Amplification was then performed using a Veriti™ 96-Well Fast Thermal Cycler (Cat. No. 4375305; Applied Biosystems™) with the following settings:

| | | |
|---|---|---|
| 1 cycle | 98°C | 30 seconds |
| | 98°C | 10 seconds |
| 30 cycles | 65°C | 30 seconds |
| | 72°C | 3 minutes |
| 1 cycle | 72°C | 7 minutes |

Once complete, the samples were stored at 4°C when not in use. To check the size of the amplified fragment(s), the products were run on a 1.2% Tris-acetate-EDTA (TAE) agarose gel in TAE buffer at 110 V for 1.5 hours. The gel was then imaged using a Axygen Gel Documentation System (GD-1000; Corning) with UV transillumination.

Following successful PCR amplification demonstrating the amplification of fragments of the size expected, we prepared samples for Sanger sequencing. To do so, PCR amplification was performed exactly as described for the initial PCR amplification, but with 100 µL reactions to increase the final concentration of the amplified DNA fragments. The products were then run on a 1.2% Tris-acetate-EDTA (TAE) agarose gel in TAE buffer at 110 V for 1.5 hours. Once complete, the distinct bands were excised from the gel using a UV transilluminator. DNA was purified from the gel fragments using a QIAquick Gel Extraction Kit (Cat. No. 28704; QIAGEN), following the manufacturer's instructions. The concentration of the purified DNA was checked using a Qubit Broad Range (BR) DNA Assay Kit (Q32850) and an Invitrogen Qubit 4 Fluorometer. Samples were then prepared for Sanger sequencing by combining 10 ng of DNA, 3 µL of 10 µM primers, and water to volume (for 12 µL samples). Three fragments were submitted to the MSU Genomics

Core for Sanger Sequencing: (1) a ~294 bp band from MWT, (2) a ~294 bp band from MWB, and (3) a ~3901 bp band from MWB. These fragments were Sanger sequenced using an Applied Biosystems 3730xl Genetic Analyzer using both VvSCD1 primers for two separate runs per fragment. The Sanger sequencing data was viewed using SnapGene Viewer 6.0 and Benchling (2023).

**Investigating GSVIVG01008260001 insertion sequence**

To see if the 3.6 kbp insertion within GSVIVG01008260001 in Merlot WB showed sequence similarities to transposable elements (TEs), we first added a contig to the 12X.v2 grapevine reference assembly that contained the insertion as well as 50,000 base pairs upstream and downstream of the insertion site. We then ran EDTA v1.9.4 on this modified fasta file with the following flags: *--species others, --step all, --overwrite 1, --sensitive 1, --anno 1, --evaluate 0*, and *--force 1*. EDTA produced a gff file with the coordinates for high confidence TEs, and we looked into the TEs annotated within the 3.6 kbp insertion sequence.
