# Supplementary material for: From buds to shoots: insights into grapevine development from the Witch’s Broom bud sport
Source: BMC Plant Biol. 2024 Apr 16;24:283. doi: 10.1186/s12870-024-04992-y (PMC11020879; doi:10.1186/s12870-024-04992-y)
Supplement: Supplementary file 8 — Supplementary Material 8 [file 12870_2024_4992_MOESM8_ESM.pdf]

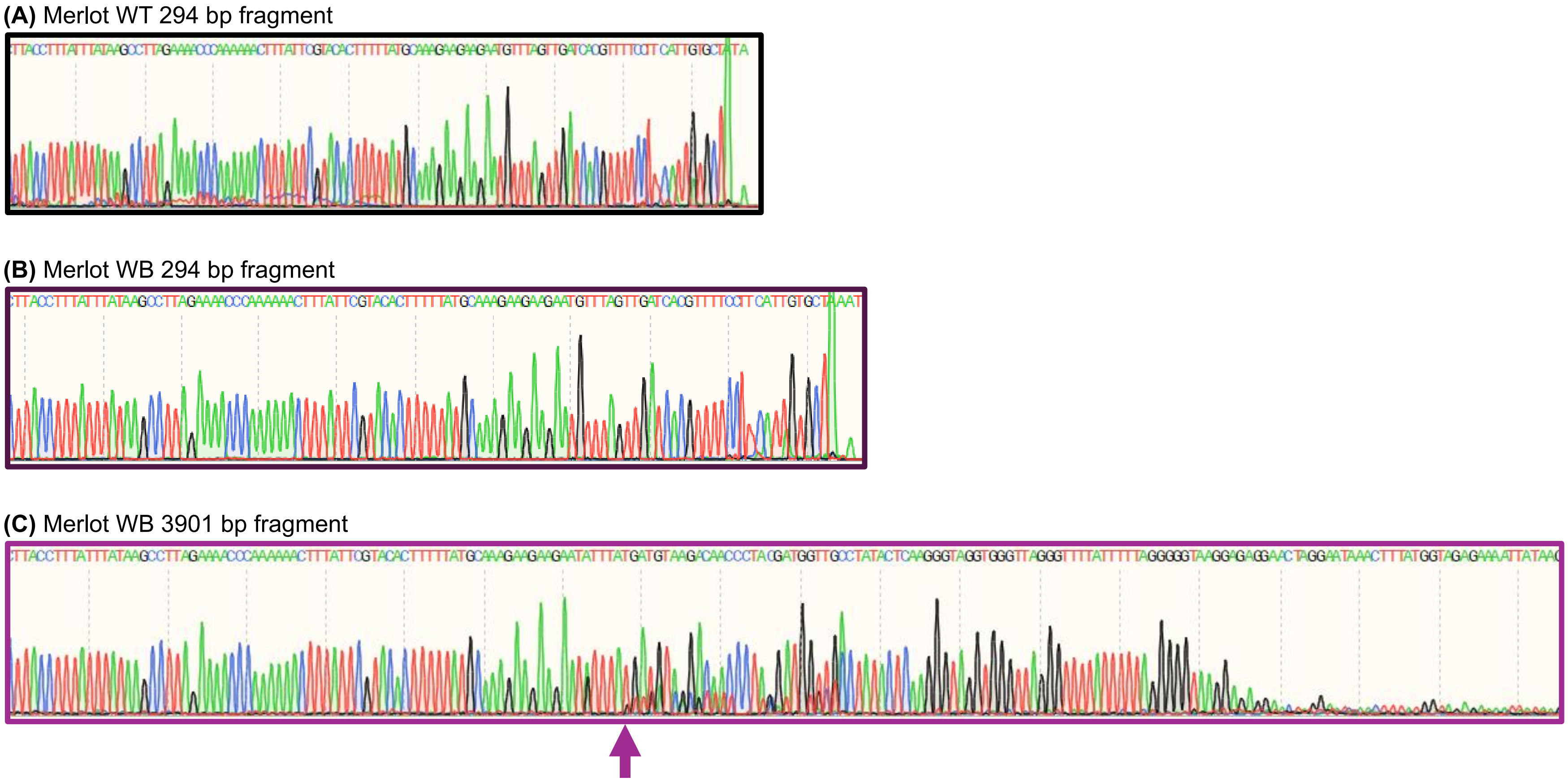

Figure S8. Sanger sequencing data of purified fragments from the region around the insertion within GSVIVG01008260001 for the 294 bp fragments amplified in **(A)** Merlot WT, **(B)** Merlot WB, as well as the **(C)** 3901 bp fragment in Merlot WB. Sanger sequencing data generated using the reverse VvSCD1 primer are shown. The sequences shown all start at identical locations within WT sequence and end at the end of the sequence generated through Sanger sequencing. The purple arrow shows approximately where the insertion sequence begins in the Merlot WB 3901 bp fragment.
