# Supplementary material for: From buds to shoots: insights into grapevine development from the Witch’s Broom bud sport
Source: BMC Plant Biol. 2024 Apr 16;24:283. doi: 10.1186/s12870-024-04992-y (PMC11020879; doi:10.1186/s12870-024-04992-y)
Supplement: Supplementary file 9 — Supplementary Material 9 [file 12870_2024_4992_MOESM9_ESM.pdf]

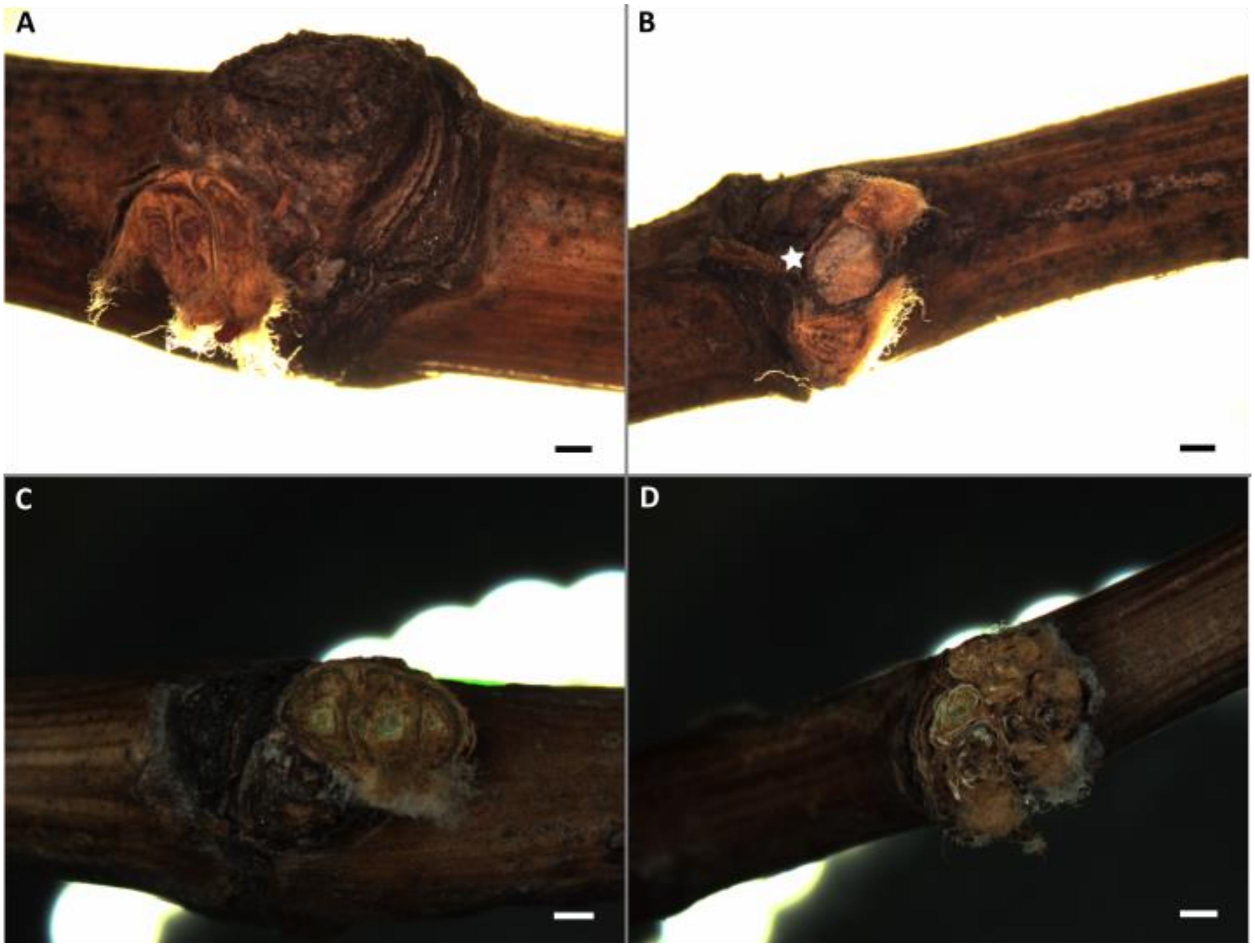

Figure S4. Buds imaged using a dissecting microscope for **(A)** Dakapo WT, **(B)** Dakapo WB, **(C)** Merlot WT, and **(D)** Merlot WB samples. The vascular tissue projecting out of the Dakapo WB sample is directly right of the solid star symbol. The scale bars are 1 mm wide.
