# Supplementary material for: From buds to shoots: insights into grapevine development from the Witch’s Broom bud sport
Source: BMC Plant Biol. 2024 Apr 16;24:283. doi: 10.1186/s12870-024-04992-y (PMC11020879; doi:10.1186/s12870-024-04992-y)
Supplement: Supplementary file 10 — Supplementary Material 10 [file 12870_2024_4992_MOESM10_ESM.pdf]

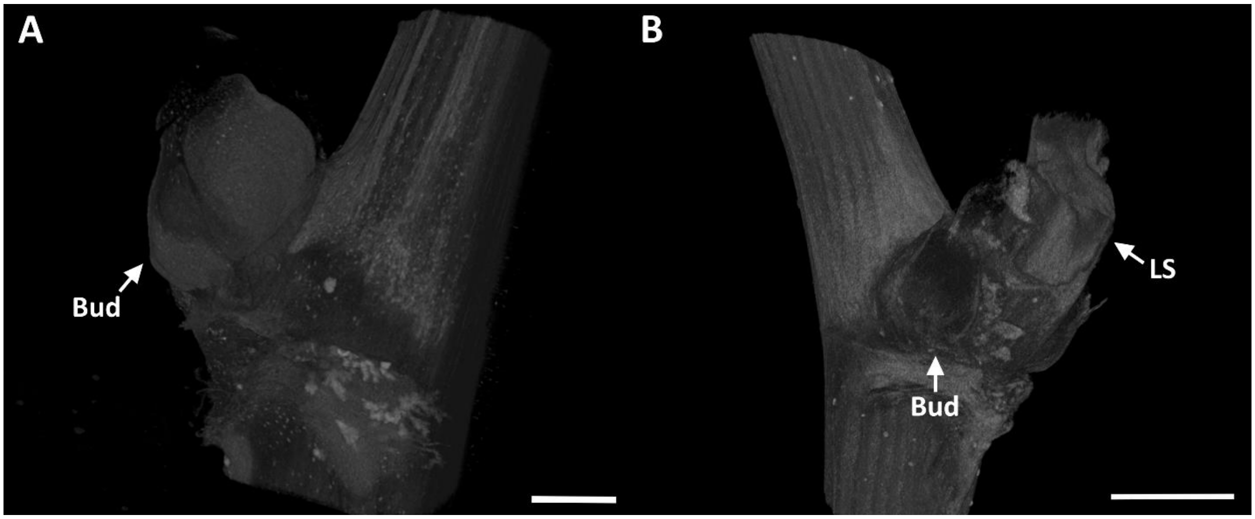

Figure S6. CT scans of **(A)** Dakapo WT and **(B)** Dakapo WB stems externally. The buds for both samples are labeled, as well as the initiated lateral shoot stem (LS) in Dakapo WB. In the Dakapo WB sample, an additional bud is present on the other side of the LS but obscured. Scale bar = 2 mm.
