# Supplementary material for: From buds to shoots: insights into grapevine development from the Witch’s Broom bud sport
Source: BMC Plant Biol. 2024 Apr 16;24:283. doi: 10.1186/s12870-024-04992-y (PMC11020879; doi:10.1186/s12870-024-04992-y)
Supplement: Supplementary file 11 — Supplementary Material 11 [file 12870_2024_4992_MOESM11_ESM.pdf]

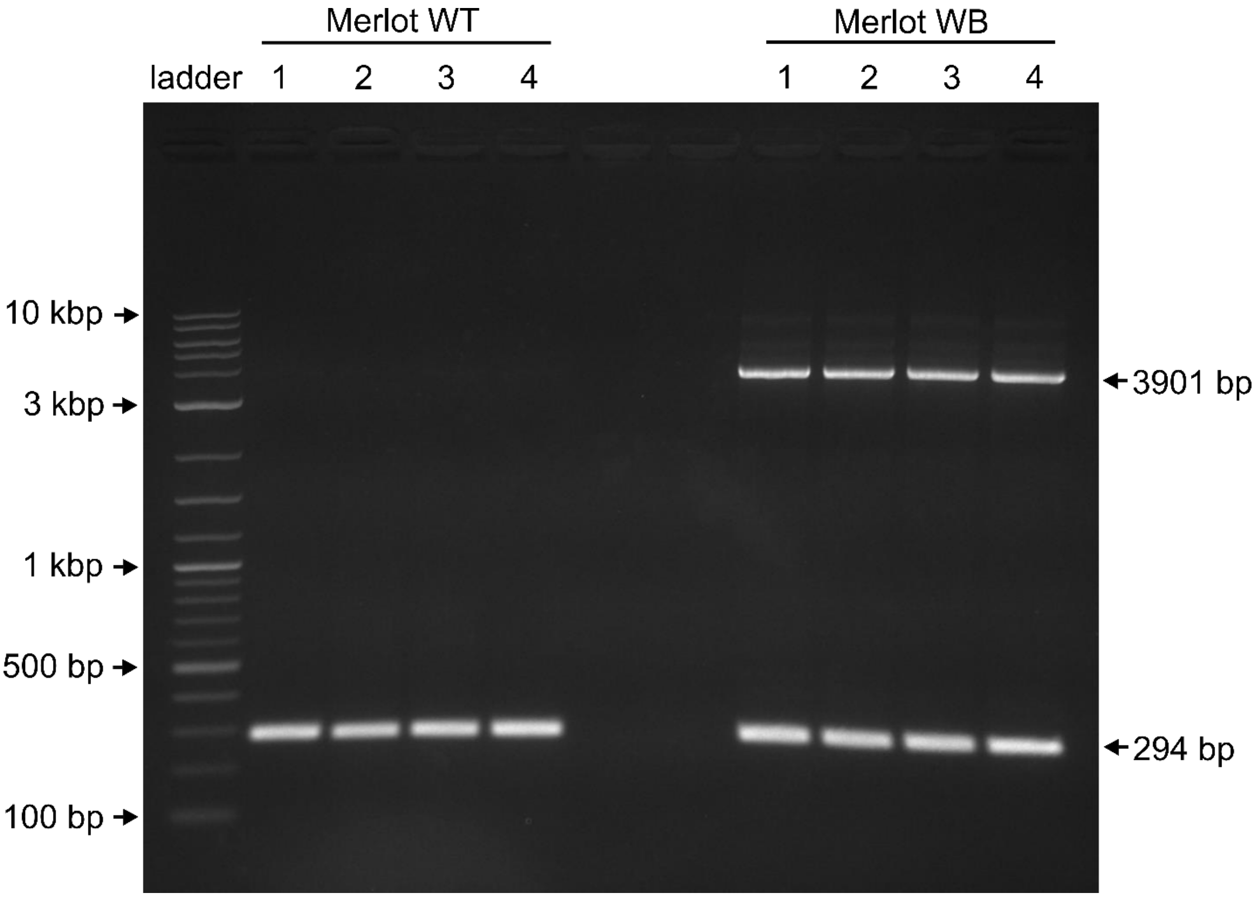

Figure S7. Agarose gel (1.2%) electrophoresis of PCR-amplified products using VvSCD1 primers. The ladder lane contains the 1 Kb Plus DNA ladder (NEB). Four technical replicates were run for each sample and were loaded into individual lanes. The amplified wild-type sequence is expected to be 294 bp in length, while the amplified WB sequence with the 3.6 kbp insertion present is expected to be 3901 bp in length. The Merlot WT sample only had bands present at 294 bp. The Merlot WB sample had two bands, one at 294 bp and one at 3901 bp, demonstrating that it is heterozygous for the 3.6 kbp insertion. Sanger sequencing of these individual DNA fragments confirmed that the bands at 294 bp were the amplified wild-type sequence of GSVIVG01008260001. Sanger sequencing also confirmed that the bands at 3901 bp were the amplified sequence of the wild-type sequence as well as the 3.6 kbp insertion within the wild-type sequence.
