# Supplementary material for: From buds to shoots: insights into grapevine development from the Witch’s Broom bud sport
Source: BMC Plant Biol. 2024 Apr 16;24:283. doi: 10.1186/s12870-024-04992-y (PMC11020879; doi:10.1186/s12870-024-04992-y)
Supplement: Supplementary file 12 — Supplementary Material 12 [file 12870_2024_4992_MOESM12_ESM.pdf]

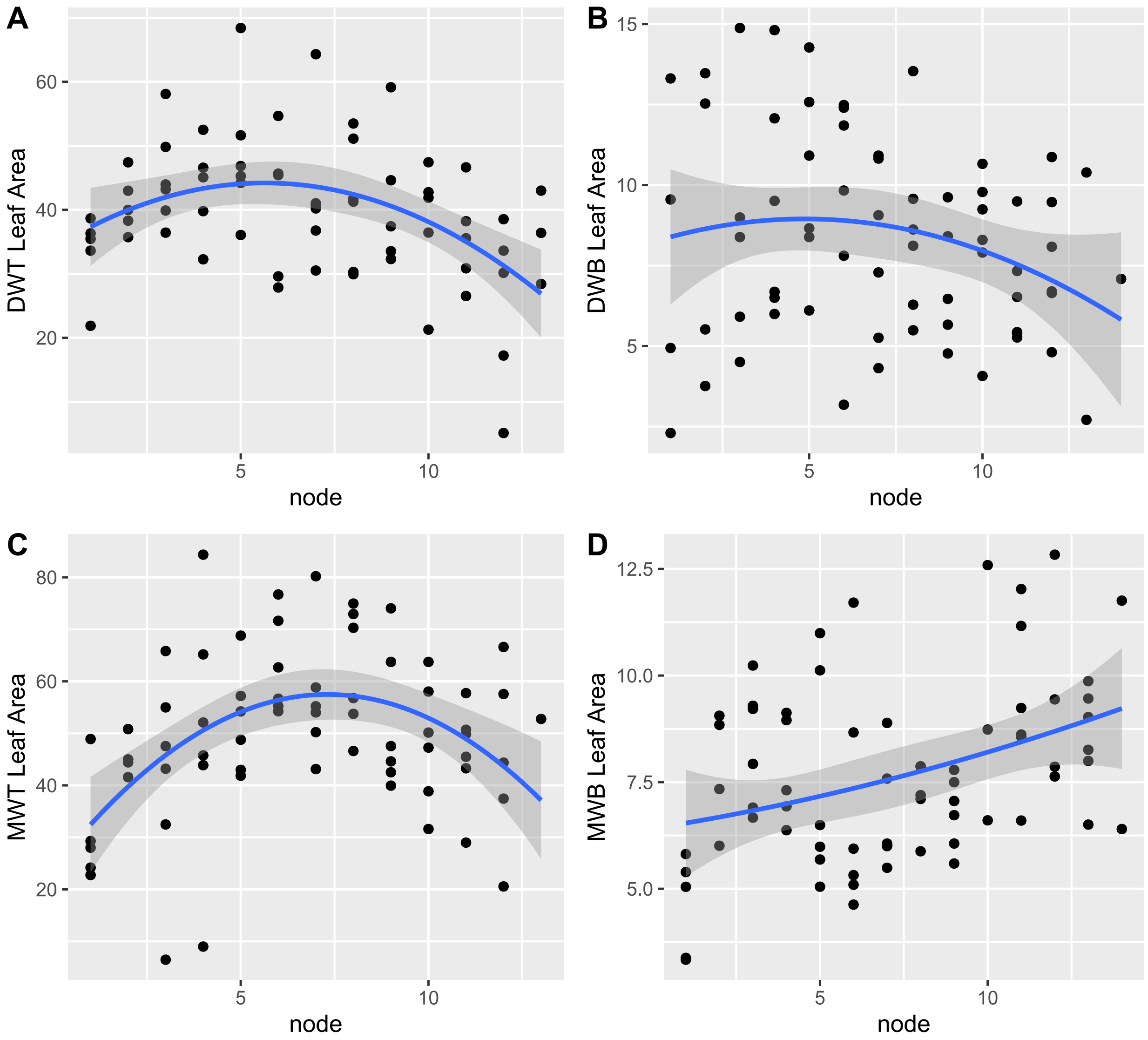

Figure S10. The developmental trajectories of leaf area across shoots for **(A)** Dakapo WT, **(B)** Dakapo WB, **(C)** Merlot WT, and **(D)** Merlot WB. The blue line represents the linear model of the formula $y \sim x + x^2$, in which y is leaf area and x is node position. There was significant support for this negative quadratic relationship between leaf area and node in both Dakapo WT and Merlot WT ($P < 0.05$ for both x and x^2 components for both varieties). However, there is not significant support for a negative quadratic relationship between leaf area and node in both Dakapo WB and Merlot WB ($P > 0.05$ for both x and x^2 components for both varieties).
