# Supplementary material for: From buds to shoots: insights into grapevine development from the Witch’s Broom bud sport
Source: BMC Plant Biol. 2024 Apr 16;24:283. doi: 10.1186/s12870-024-04992-y (PMC11020879; doi:10.1186/s12870-024-04992-y)
Supplement: Supplementary file 13 — Supplementary Material 13 [file 12870_2024_4992_MOESM13_ESM.pdf]

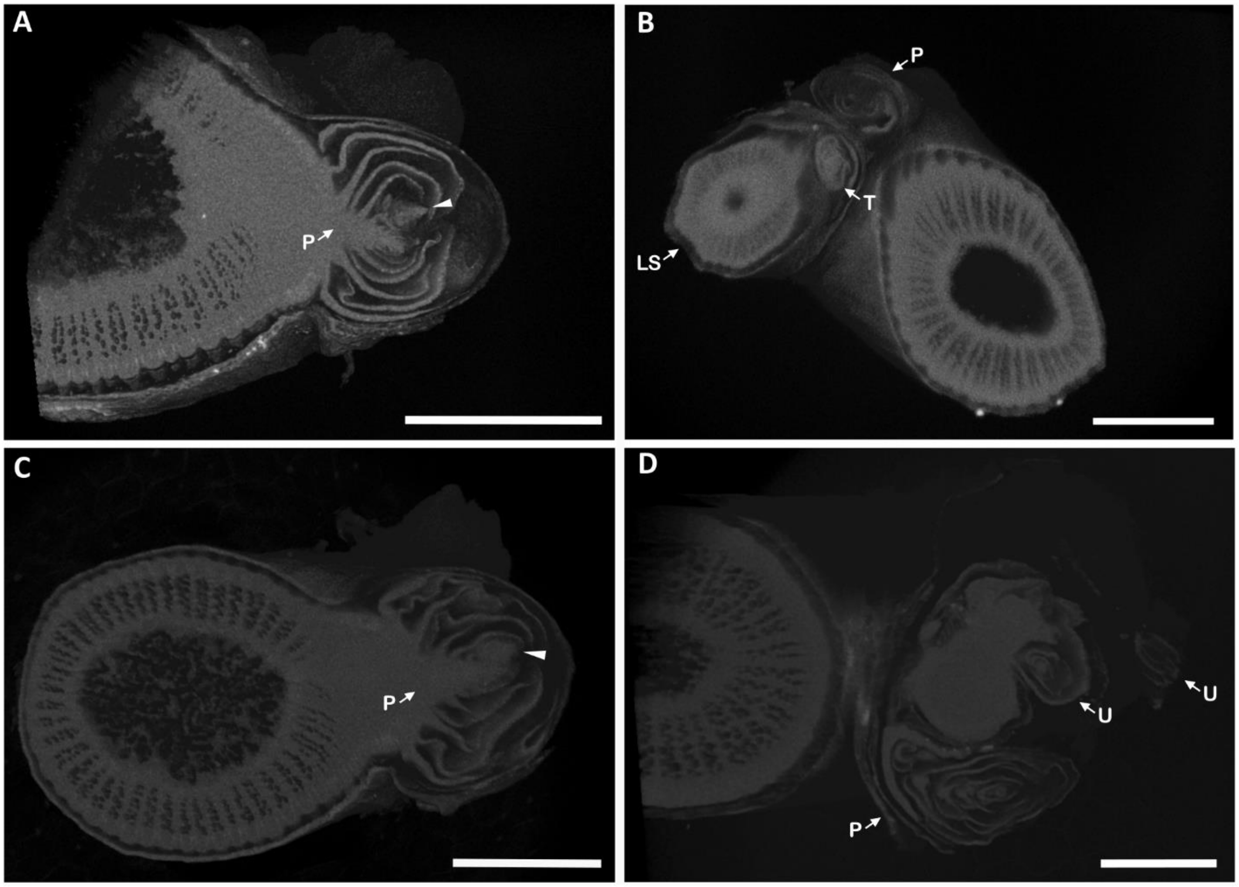

Figure S5. CT scans top-down of buds from **(A)** Dakapo WT, **(B)** Dakapo WB, **(C)** Merlot WT, and **(D)** Merlot WB samples. Primary primordia (P) are labeled in all four samples. The inflorescence primordia are indicated by the solid triangle in the **(A)** Dakapo WT and **(C)** Merlot WT samples. The tertiary primordium (T) and the lateral shoot stem (LS) are indicated in **(B)** Dakapo WB and the uncharacterized primordia (U) in **(D)** Merlot WB are indicated as well. Additional bud primordia are present in all samples but obscured due to the angle of the images. Additional inflorescence primordia are present in the WT samples but obscured in the images as well. Scale bar = 2 mm.
