# Supplementary material for: From buds to shoots: insights into grapevine development from the Witch’s Broom bud sport
Source: BMC Plant Biol. 2024 Apr 16;24:283. doi: 10.1186/s12870-024-04992-y (PMC11020879; doi:10.1186/s12870-024-04992-y)
Supplement: Supplementary file 16 — Supplementary Material 16 [file 12870_2024_4992_MOESM16_ESM.pdf]

	Dakapo		Merlot	
	**WT**	**WB**	**WT**	**WB**
Novel SNVs	349,533	349,239	351,018	356,754
Novel INDELs	61,075	62,186	58,570	62,064

Table S6. Novel* SNVs and INDELs for all four samples.

*Novel variants are variants completely absent in the sample of the same variety
